# Peers, parents, and self-perceptions: the gender gap in mathematics self-assessment

**DOI:** 10.1007/s00148-025-01087-2

**Published:** 2025-02-22

**Authors:** Anna Adamecz, John Jerrim, Jean-Baptiste Pingault, Nikki Shure

**Affiliations:** 1 UCL Social Research Institute, KRTK KTI and IZA, London, England; 2 UCL Social Research Institute, London, England; 3 UCL Department of Clinical, Educational and Health Psychology and KCL Social, Genetic & Developmental Psychiatry Centre, London, England; 4 UCL Social Research Institute and IZA, London, England

**Keywords:** Gender gaps, Self-assessed mathematics ability, Twins, Peer effects, I24, J16

## Abstract

It is well established that boys perceive themselves to be better in mathematics than girls, even when their ability is the same. We examine the drivers of the gender gap in self-assessed mathematics ability using a longitudinal study of twins. Using measures of individual self-assessment in mathematics from childhood, along with mathematics levels and test scores, cognitive skills, parent and teacher mathematics assessments, and characteristics of their families and siblings, we examine potential channels of the gender gap. Our results confirm that objective mathematics abilities only explain a small share of the gender gap in self-assessed mathematics abilities, and the gap is even larger within boy-girl twin pairs. We find that the self-assessment of boys is positively correlated with the self-assessment of their male co-twins, not just in mathematics, but also in other abilities. However, this positive correlation is not observed between girls and their male co-twins; if anything, it is negative. This indicates that boys and girls have different reactions to highly confident male peers. We also find that parents are more likely to overestimate boys’ and underestimate girls’ mathematics abilities. Gender-biased parental assessments explain a large part of the gender gap in mathematics self-assessment, highlighting the potential of the intergenerational transmission of gender stereotypes.

**Supplementary Information:**

The online version contains supplementary material available at 10.1007/s00148-025-01087-2.

## Introduction

Across a range of countries, contexts, and domains, men have been found to exhibit higher degrees of confidence in their ability than women (Kay and Shipman 2014). This phenomenon has been particularly salient in the fields of science, technology, engineering, and mathematics (STEM). Not only do girls assess their mathematics ability lower than boys from an early age (Baird and Keene 2019), but this contributes to later gender gaps in mathematics performance (Bharadwaj et al. 2016) and disparities in pay (Sterling et al. 2020). This is important since mathematics skills and participation and success in STEM fields have been linked to high labor market returns (Walker and Zhu 2011).

**Fig. 1:**
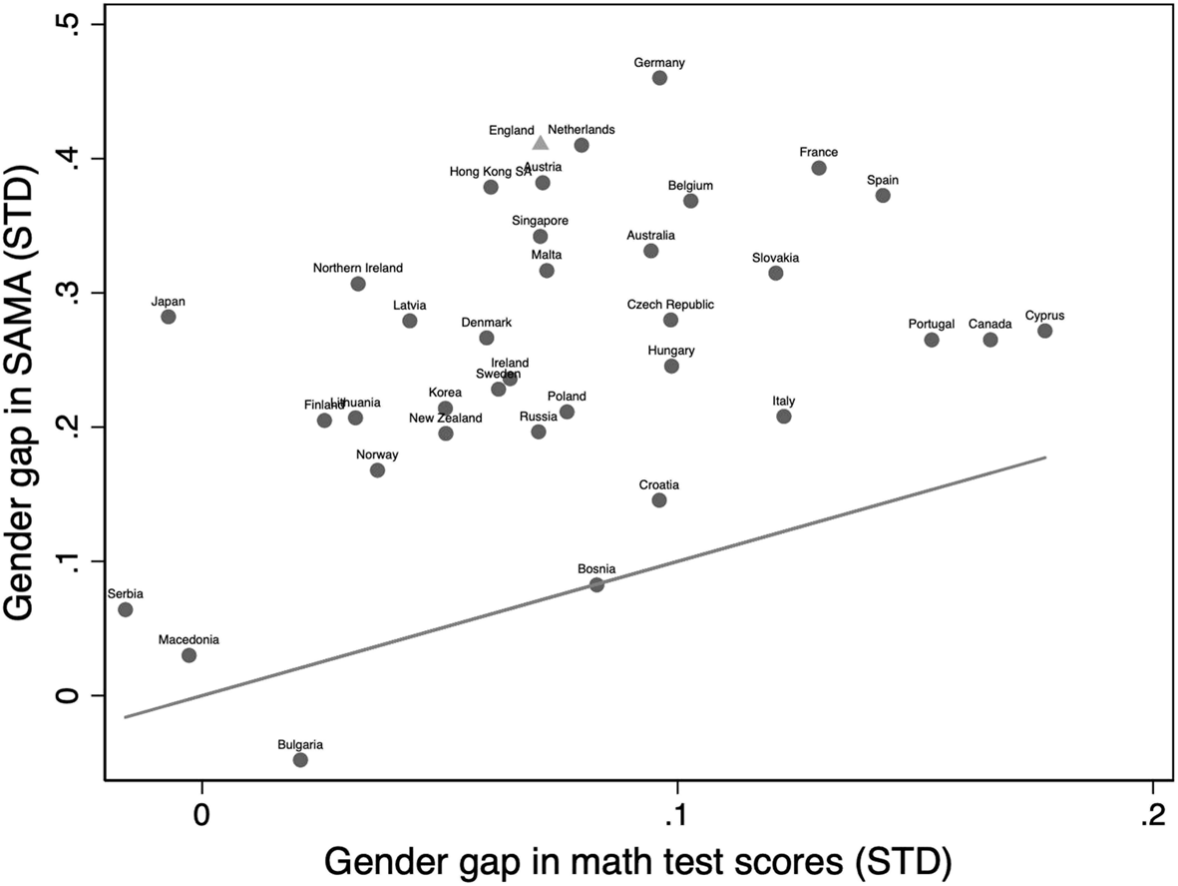
The gender gap in mathematics test scores and self-assessed mathematics ability (SAMA) internationally. *Notes:* SAMA and mathematics test scores have been standardized to mean zero and standard deviation one. The gender gap is calculated as the average boys’ score minus the average girls’ score within each country. A positive gap, therefore, denotes a gender gap in favor of boys. The 45° line indicates the theoretical equality of the gender gap in SAMA and in mathematics performance; in countries above the line, the gender gap in SAMA is larger than the gender gap in mathematics performance. Source: TIMSS, Grade 4 (2019)

Although the gender gap in mathematics performance (both grades and test scores) is narrowing in many countries, the gender gap in the self-assessment of mathematics abilities (SAMA) is still much larger. Figure 1 highlights this phenomenon using data from the most recent wave of the large-scale international assessment, Trends in International Mathematics and Science Study (Mullis et al. 2020). Almost all countries are above the 45° line, indicating that the gender gap in favor of boys is larger in SAMA than in mathematics performance; the magnitude of the difference in mathematics performance ranges from 0 to 0.2 standard deviations while the difference in self-assessed mathematics ability ranges from 0 to 0.45 standard deviations.

While the gender gap in mathematics performance has received much scholarly attention (e.g., Fryer and Levitt 2010), less has been paid to the drivers of the gender gap in SAMA. Of course, the two are related, since individuals who are good at something tend to also rate their ability highly. What is perhaps worrying, however, is that the gender gap in favor of men in self-assessed ability has been shown to remain even between individuals of the same ability or when women outperform men (Ehrlinger and Dunning 2003; Niederle and Vesterlund 2007). This male overconfidence in their ability has been shown to explain later inequality in the labor market (Adamecz-Völgyi and Shure 2022). Trying to understand the drivers of the gender gap in self-assessed mathematics ability is therefore important.

This paper explores the drivers of the gender gap in SAMA during childhood and adolescence. We use a longitudinal study of twins from the UK that allows us to control for otherwise unobserved heterogeneity in the genetic factors, family background, and environment of boys and girls without birth order or age effects. Exploiting the rich nature of the data, we estimate the gender gap in SAMA at age nine and age 12 using linear regressions conditioning on actual mathematics ability as well as a range of individual, twin pair, and family characteristics. We draw on existing literature from education, psychology, and economics to explore the potential channels of the gender gap.

We make three contributions to the literature. First, we show that the gender gap in SAMA persists even after controlling for mathematics grades given by teachers, mathematics test scores, measures of verbal and non-verbal cognitive abilities, birth order, birth weight, and twin fixed effects, i.e., shared genetic and environmental context. Importantly, objective skills only explain 14–26% of the gender gap in SAMA. We document a similar gender gap in the parental assessments of children’s mathematics performance, as well as in teachers’ assessments, although the latter is smaller.

Second, we show that the gender gap in SAMA is even higher among boy-girl twins than among non-related boys and girls (boy-boy and girl-girl twin pairs). We find the gender gap in parental assessments of mathematics ability higher among boy-girl twins, even when we control for the twins’ mathematics ability. These results suggest that within boy-girl twin pairs, there might be a stronger emphasis on who is the “mathematics person” (the boy) and the “verbal person” (the girl) within the family. This differentiation is captured in the assessments of parents and might hurt girls’ confidence in their mathematics ability.

Third, we test three potential channels of the gender gap in SAMA, which emerge from the literature: (1) twin peer effects; (2) parental and teachers’ assessments in general, and stereotypically gender-biased parental assessments in particular; and (3) the comparative advantage of girls in English relative to math. We provide further details on these channels in the next section.

In terms of peer effects, we find that having a male co-twin (as opposed to a female co-twin) is negatively correlated with SAMA, for both boys and girls alike. We do not find a significant relationship between having a male non-twin sibling on average, although for girls, the magnitude of the negative relationship between having a brother (who is not their co-twin) and SAMA is about the same as the relationship between SAMA and having a male co-twin. This highlights the importance of frame-of-reference or contrast effects for girls.

Interestingly, the mathematics performance of one’s male co-twin does not contribute to the gender gap in SAMA. The SAMA of the male co-twin, however, matters, and this relationship is gender-specific. The confidence of boys is positively correlated with the confidence of their male co-twin, while between girls and their male co-twins, this positive correlation is not present. If anything, for a girl with a male co-twin, the more confident her brother is in his mathematics abilities, the less confident she is. In other words, having a confident male co-twin seems to only be good for boys. This is true not only in mathematics but also in English (where girls perform better and exhibit higher confidence than boys) and in physical abilities (where boys are slightly more confident). These results could indicate that some of the educational and labor market gender gaps, like those in STEM studies and top jobs, might be related to this phenomenon. STEM tracks and top jobs are traditionally filled by confident men, and such a peer group might make entry easier for men. An important caveat of these results is that one’s co-twin’s self-assessment is not random.

While we are not able to identify the causal effect of parental evaluations on SAMA, we find suggestive evidence that the intergenerational transmission of gender stereotypes might be important in producing the gender gap in SAMA. As mentioned above, parents also exhibit a gender bias when assessing their sons’ and daughters’ mathematics abilities. Even teachers exhibit a similar bias in how they assess male and female pupils. Parental assessments make a large contribution to the gender gap in SAMA: they explain 23% of the gap even when we account for the twins’ actual mathematics ability. We probe this channel further by constructing a binary variable that captures whether the assessment of parents is stereotypically gender-biased, i.e., they underestimate their daughter or overestimate their son in math. We find that the largest gender gap in SAMA is among those young people with stereotypical parental assessments.

In terms of comparative advantage, we find that although those with higher performance in English have lower SAMA (hence, they are more likely to view themselves as a “verbal person”), this relationship is not gender-specific; thus, it does not contribute to the gender gap in SAMA. It is true for both genders that their (conditional) self-assessment in mathematics is positively correlated with their self-assessment in English, and this correlation is even higher for girls. This result suggests that general confidence in abilities might be more important for girls in terms of how they self-assess their mathematics ability.

Taken together, our results lend support for the transmission of gender biases from adults to children, and from male peers to men, even though we cannot supply causal evidence in this respect. We suggest that potential interventions aiming to increase SAMA among girls and decrease the gender confidence gap, in general, should also target parents. Furthermore, as we also document a gender gap in teachers’ assessments, conditional on mathematics levels that they themselves gave to their students, we suggest increasing teachers’ awareness of their potentially gender-biased performance evaluations.

The rest of the paper is structured as follows. In Sect. 2, we elaborate on the potential channels of the gender gap in SAMA outlined in the introduction. In Sect. 3, we present the data used in this paper as well as some descriptive statistics. In Sect. 4, we outline the empirical strategy. This is followed by the results of our estimation in Sect. 5. Finally, in Sect. 6, we conclude.

## Related literature and potential mechanisms

In order to understand the gender gap in how boys and girls assess their mathematics performance, it is necessary to start by examining the gender gap in actual mathematics performance. There is a gender gap in favor of boys in mathematics performance across ages in most countries. This gap emerges in primary school and widens by adolescence and early adulthood (Borgonovi et al. 2021; Bedard and Cho 2010). By age 15, results from the Programme for International Student Assessment (PISA) show a gender gap in favor of boys across a range of countries, with the gap largest in developed countries (Bharadwaj et al. 2016). The magnitude of these gaps can be equated to girls undergoing one to four months less of schooling depending on the country (Woessmann 2016). The gender gap in mathematics in favor of boys also appears on high-stakes tests such as the SAT (approximately 0.3 SD over a 40-year period) or the GRE in the USA (Brown and Pinel 2003; Fryer and Levitt 2010)).

Trying to understand the explanation behind the gender gap in mathematics performance has produced a range of literature. This literature has tended to differentiate between biological differences (e.g., Wilder and Powell 1989) and societal explanations (e.g., Guiso et al. 2008). Recent advancements in neuroscience have ruled out a biological explanation for this difference (Rippon 2020) since much of brain development seems to be shaped by the social environment. This highlights the importance of social explanations for gender differences in mathematics performance, including the importance of gender stereotypes. Gender stereotypes are pervasive and may shape a range of behaviors, which in turn shape the gender gap in mathematics performance. This includes lower investment by girls in math, low parental expectations, and biased tests; however, Fryer and Levitt (2010) do not find support for these hypotheses in explaining the gender gap in mathematics performance. Instead focus has been placed on how stereotypes influence cultural contexts (Guiso et al. 2008; Nollenberger et al. 2016), gendered behavior (Niederle and Vesterlund 2010), and teachers (Carlana 2019), all of which explain the gender gap in mathematics performance. In societies where gender stereotypes are more pervasive and gender equality indices are lower, the gender gap in PISA is larger (Guiso et al. 2008). Niederle and Vesterlund (2010) show that the competition associated with test-taking puts women at a disadvantage, but actually increases men’s performance. Carlana (2019) examines how teachers’ implicit bias, based on gender stereotypes, is transmitted to students and hurts girls’ performance in STEM.

We build on this previous literature by exploring the gender gap in SAMA. SAMA is important because it is a measure of self-perception, much like self-concept, which has been shown to positively shape future life outcomes (Marsh and Yeung 1997; Hansen et al. 2023). “Self-concept” is defined as “a person’s self-perceptions that are formed through experience with and interpretations of one’s environment” (Marsh et al. 2012). More generally, self-concept can be defined by an individual’s perception of their competency in a specific activity (Wigfield and Eccles 2000). Individuals who view their mathematics ability favorably and have high academic self-concept in mathematics are more likely to achieve success in mathematics and pursue STEM fields in school and university (Marsh 1990). As STEM occupations are the highest paid in many countries, including the UK (Walker and Zhu 2011; Henderson et al. 2020), this occupational sorting is an important driver of the gender wage gap. This means that understanding gender gaps in SAMA can translate into better understanding gender gaps across labor market outcomes, which is desirable from a policy perspective.

The gender gap in SAMA has been established in early age (Baird and Keene 2019; Bharadwaj et al. 2016) and is a stylized fact across a range of countries (see Fig. 1). Given the difference in magnitude across countries participating in TIMSS between the gender gap in mathematics performance (approximately 0.1SD) as compared to the gender gap in SAMA (approximately 0.4SD), it is worthwhile understanding why. Many of the potential explanations behind this gender gap are related to the gender gap in mathematics performance and also draw on societal explanations. As in the literature on the gender gap in mathematics performance, we focus on the role of stereotypes and how they impact: social interactions with the closest peer, one’s twin; the transmission of parents’ and teachers’ assessments; and the self-identification as a “mathematics person.” By examining these three levels (co-twin, adults, and self), we can begin to better understand how gender stereotypes shape the gender gap in SAMA. Gender stereotypes seem particularly salient in this context where the gender gap in SAMA is much larger than the gender gap in actual mathematics performance.

The social environment in which children interact with each other is arguably shaped by gender stereotypes. There is an extensive literature on peer effects in economics (Sacerdote 2011), including siblings (e.g., Nicoletti and Rabe 2019), and an individual’s twin is likely to be their main point of reference or comparison (i.e., their key peer). Girls with brothers have a boy as their closest peer and the most direct point of comparison. Experimental literature shows that women shy away from competition (Niederle and Vesterlund 2007), which means that girls with brothers may shy away from mathematics and identifying as the “mathematics” person if they perceive this role to already be taken by their brother. We would not expect this to be true of girls with sisters.

Peer effects may also manifest via differential parenting by gender, which could arise for a variety of reasons. Parents may have set gender roles within the home that reinforce societal gender stereotypes. It has been shown that growing up in families with a preference for sons decreases girls’ mathematics performance (Dossi et al. 2021), which may be related to the time that parents invest in helping sons with their mathematics, but not girls. In this case, girls with twin brothers may again not identify as the “mathematics person” since their parents may already assign this role to their brother. In the case of two twin boys, both might vie for this role without the issue of parental stereotypes.

We investigate these peer effects looking at the gender-specific correlation between one’s own SAMA and their co-twin’s mathematics ability and SAMA. These variables are interacted with gender to explore heterogeneous effects by gender. The hypothesis here is that exposure to a “math person,” whether measured by actual or self-assessed ability, as the closest peer may discourage girls from identifying as a “math person” themselves, especially if that closest peer is a boy since this follows gender stereotypes.

Having a brother as a twin may also have a biological impact. There is a literature examining the long-term effects of in-utero testosterone exposure (Auyeung et al. 2009). Bütikofer et al. (2019), for example, find that women exposed to increased testosterone in-utero via a male twin experience a lower probability of completing education and lower fertility later in life. This also holds true for women whose male twin died shortly after birth, indicating the importance of this biological channel over and above the environmental channel of growing up with a brother as one’s closest peer. This biological exposure to increased testosterone could translate into lower SAMA just as it decreases the probability of completing schooling. We are not able to disentangle the biological channel from the environmental channel here, but this negative impact of testosterone exposure would be picked up through the inclusion of a male twin variable.

Psychologists have pointed to the importance of gender stereotypes, where certain fields are viewed as either feminine or masculine, in determining how individuals assess their own ability in those subjects. This has its origins in social role theory, which states that gender stereotypes emerge because we observe men or women occupying certain positions in society (Eagly and Wood 2012). There is well-documented evidence that both men and women view mathematics as a masculine subject (Makarova et al. 2019). This implies that girls may self-assess their mathematics ability lower than boys because they learn these biased assessments from the adults (e.g., teachers and parents) in their environment. When these adults are particularly gender stereotypical in how they assess children, their assessments may be even more salient. In a related paper using Australian data, Nicoletti et al. (2022) show that parents assess sons’ mathematics ability higher than daughters’.

We explore this channel by including parental and teacher assessments of the children’s mathematics ability in our models. How young people interact with parents and teachers will also be shaped by gender stereotypes and norms. All of the children in our sample live in the UK, so we cannot exploit country-level differences in gender equality, but we can differentiate by family context, i.e., the fact that some families will be more gender equal than others. We create an indicator for whether parents assess their children’s mathematics ability according to gender stereotypes and include this in the model. This variable is also interacted with gender to explore the differential effects of gender stereotypes for boys and girls. We would expect the transmission of gender stereotypes to be especially important in more conservative households where gender roles are clearly defined based on stereotypes. By including these adult assessments, we are testing the hypothesis of whether gender stereotypes held by individuals in role model positions are related to the self-perceptions that children hold and whether this is especially salient in more gender stereotype-conforming households.

In social psychology, people are assumed to see themselves as either a “mathematics” person or a “verbal” person, but usually not both at the same time (Marsh and Hau 2004). Furthermore, results of consecutive rounds of PISA, show that boys outperform girls in mathematics, but girls are usually much better in reading than boys (OECD 2020). In PISA 2018, the average gender gap in favor of girls was six times as large in reading (30 PISA points) as the average gender gap in mathematics in favor of boys (5 PISA points). Theoretically, the comparative advantage of girls in English might enhance their self-assessment of being a verbal person rather than a mathematics person. This could in turn explain some of the gender gap in SAMA. Goulas et al. (2020) find that the comparative advantage of boys in STEM subjects relative to non-STEM subjects explains at least 12% of the gender gap in STEM specialization while Breda and Napp (2019) show that comparative advantage in mathematics explains 75% of the gender gap in math-intensive studies.

We explore the comparative advantage of girls in English hypothesis by controlling for measures of English ability as well as self-assessed English ability. These variables also are also interacted with gender to explore heterogeneous impacts. Here, we test the hypothesis that girls understand their comparative advantage and therefore their self-assessment reflects this. This comparative advantage may reflect the fact that they actually outperform boys in English and/or gender stereotypes about girls being the “verbal person” and therefore internalize this in their self-assessments.

Of course there may be alternative channels of the gender gap in SAMA that we are not able to explore due to data limitations. The social environment of the twins beyond their homes may play an important role in transmitting gender stereotypes. It would be interesting, for example, to examine peer effects in schools, but unfortunately, we do not have information as to whether the twins attend the same school or are in the same class. As there is no national policy (Goymour 2017) nor data on this, we are unable to probe this further.

## Data and descriptive statistics

We use data on twins born in the UK from the Twins Early Development Study (TEDS) (Rimfeld et al. 2019). TEDS is a longitudinal study of over 10,000 twin pairs born in England and Wales from 1994 to 1996 (in four school cohorts), who were followed from birth to the present. The original sampling frame was all twin pairs born during this period. There are currently 13 waves of data available. The data includes rich, repeated measures of cognitive and non-cognitive skills, parental background, and educational outcomes. We are aware that twin samples are not necessarily representative of the population, which might hinder the external validity of our results. The mothers of twins tend to be on average older, higher educated, and healthier than the mothers of singletons due to IVF (Bhalotra and Clarke 2019).

Nevertheless, using a twin study has advantages. TEDS offers the possibility of looking at the gender gap in SAMA while controlling for shared genetic and home environments, which would not be possible in most other birth cohort studies since they usually follow one individual over time. Even in a household longitudinal study or a cohort study of siblings, we would be concerned about how birth order effects influence parenting and other potential channels of gender gaps (e.g., differences in the schooling environment or curriculum due to changes over time).

TEDS is well suited to answer our research question and robustly estimate gender gaps in SAMA due to the measures it includes. SAMA was first collected in the age nine sweep of TEDS. At this age parents and teachers were also asked to assess the twins’ mathematics ability in the same domains^1^. In addition to SAMA, we have teacher-assessed national curriculum levels, an objective measure of mathematics ability, as well as other cognitive ability measures administered as part of the survey. It is rare in longitudinal studies to have measures of 1) individuals’ self-assessments, 2) assessments by their parents and teachers on the same skills, and 3) objective measures of their performance on those domains. Having this for twin pairs makes TEDS ideal as we are able to include family fixed effects in all our models and account for shared genetics and environment.

The age nine data collection was restricted to twins born between January and August 1994 (Cohort 1) and twins born between September 1994 and August 1995 (Cohort 2). Our main estimation sample includes those who have non-missing data for the variables we use at age nine (3877 individuals). This is a rather small sub-sample of the main study (15,216 individuals in Cohort 1 and 2) because we require data for both twins as well as data from their parents and teachers.

We investigate how this sub-sample of TEDS relates to those who either dropped out or did not provide all data that we need at age nine (11,339 individuals) in Table O1 in the Online Appendix. Furthermore, we provide robustness checks to our main results in the Online Appendix where we account for the observable selection of those in our analytic sample using three methods to create weights: probit, random forest, and entropy balancing. We model the probability that individuals are included in our analytical sample using probit and random forest models. Control variables include information collected in the first wave: parental education and measures of socioeconomic status, family structure, number of siblings, and ethnicity. We fit the individual-level estimated probabilities of being in the analytical sample from both approaches and re-estimate our main results by using the inverse of these probabilities as estimation weights. As those included in the analytical sample differ from those who dropped out (or not reported data) (Table O1 in the Online Appendix), we apply a balancing technique, entropy balancing (Hainmueller 2012), to construct individual-level weights to equate the moments of the distributions of these variables across the two groups. Using these entropy-balanced weights, we weight individuals in the analytical sample in such a way that their individual characteristics have the same distribution as the individual characteristics of those who were excluded from the sample. We show in Fig. O4 in the Online Appendix that using these weights eliminates statistical differences between those in the main sample and those who were excluded. Re-estimating our (unweighted) main results using any of these three methods leads to similar results; thus, we are confident that (observed) sample selection is not driving our results.

SAMA was also collected in the age 12 sweep, which we use to provide robustness checks to our main results. Because parents and teachers were not asked to assess the twins’ ability at this age, it is not our preferred wave. We also provide a robustness check on our main model using the overlap of the age nine and age 12 samples (509 individuals).

### Self-assessed mathematics ability (SAMA)

TEDS measures self-assessed mathematics ability via three survey questions administered at ages nine and 12. The survey asks the following three questions:

*How good do you think you are at:*

1. *Solving number and money problems.*
2. *Doing Maths in your head.*
3. *Multiplying and dividing.*

There are five ordinal answers to each: very good; quite good; doing OK; not so good; not good at all, coded using a Likert scale from one (worst) to five (best). The average of responses to the three questions is provided in the data. The average SAMA at age nine is 3.83 in our analytical sample (Table A2 in Appendix A). For the purposes of our regression models, we standardize the SAMA measure to mean zero and standard deviation one so that all coefficients may be interpreted in terms of effect sizes.

**Fig. 2:**
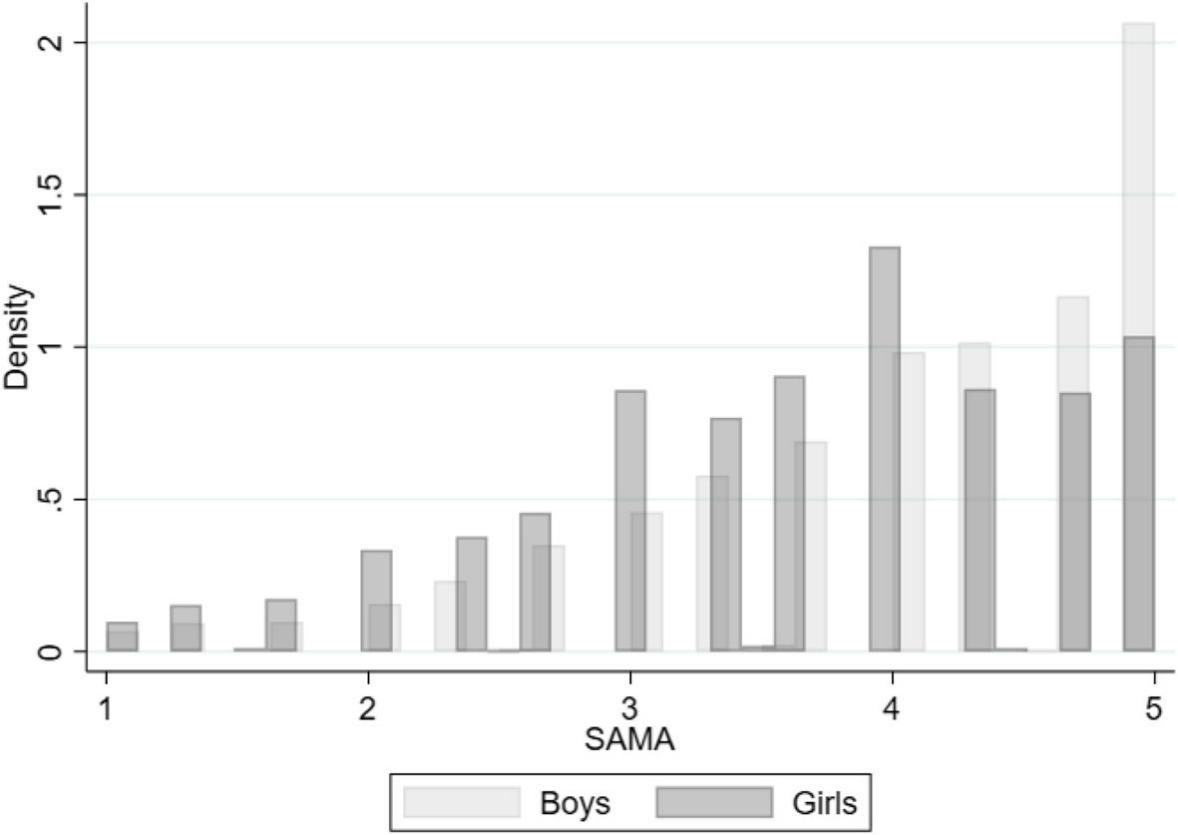
The distribution of self-assessed mathematics ability, age nine. *Notes:* *N* = 3877. Source: TEDS (Rimfeld et al. 2019). The five ordinal categories are the following: 1: not good at all; 2: not so good; 3: doing OK; 4: quite good; 5: very good

Figure 2 presents the distribution of SAMA for the age nine sample by gender. Interestingly, both distributions are shifted to the right: the majority of individuals have a positive view of their mathematics abilities. This result corresponds to findings in the overconfidence literature that people are overconfident in their ability on average (Alicke et al. 2005; Dunning et al. 2004). It is also clear that boys assess their mathematics abilities higher than girls on average. The distribution of SAMA is skewed to the right for both genders, but boys show a larger bunching at the highest self-assessment level. In our main analytical sample, the raw gender gap in SAMA at age nine is 0.382 standard deviations (Table A3 in Appendix A), which is similar in magnitude to the gender gap in TIMSS grade 4 mathematics self-concept reported in Fig. 1.

### Objective skills in mathematics

#### Mathematics levels

Teachers evaluate their students’ mathematics ability at ages seven, nine, and 12 according to National Curriculum levels (1 to 5) on three aspects of math: using and applying mathematics; numbers and algebra; shapes, space, and measures. This was used by the survey organizers to compute an overall sum score ranging from 3 to 15, which was then standardized to mean zero and standard deviation one.

**Fig. 3:**
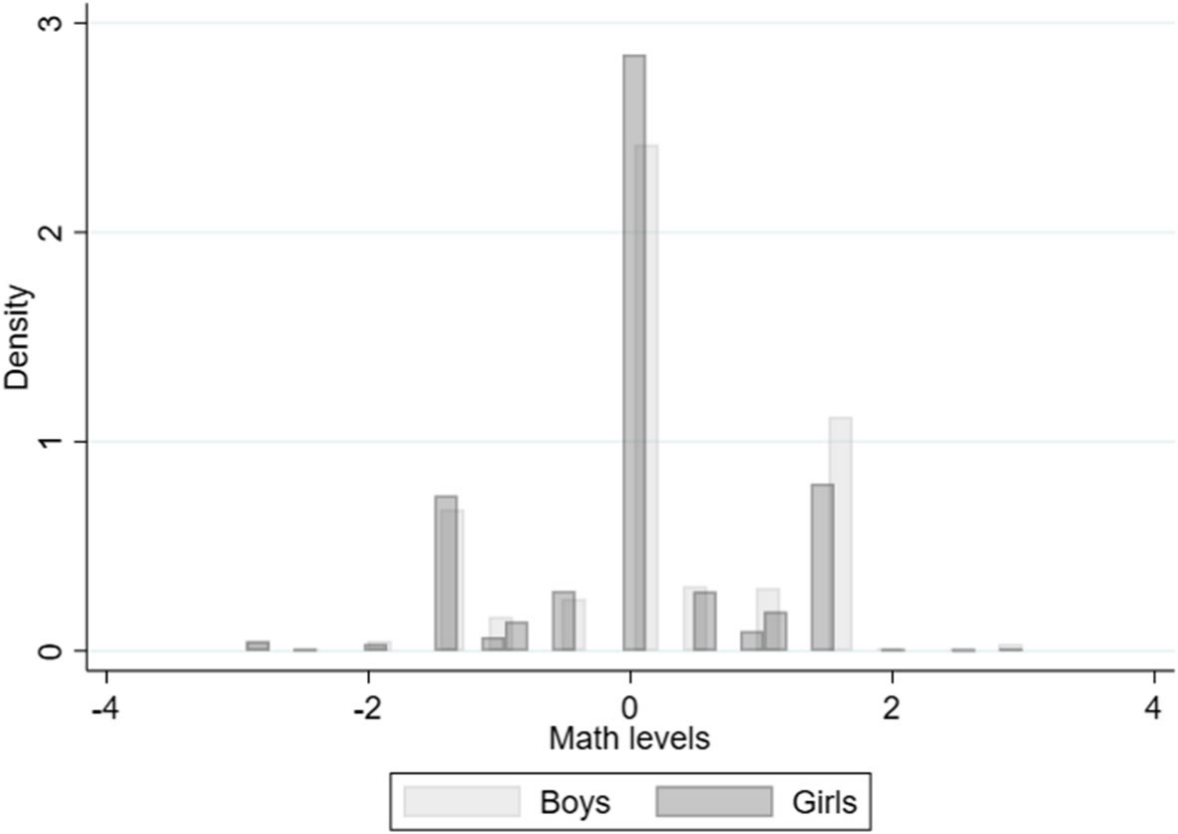
The distribution of mathematics levels, age nine. *Notes:* *N* = 3877. Source: TEDS (Rimfeld et al. 2019)

Figure 3 shows the distribution of observed mathematics ability by gender. As this measure has been standardized over the total TEDS sample, the average is zero. This figure shows that boys outperform girls in mathematics at age nine. In our analytical sample, at age nine, the mathematics level of boys (0.157) is 0.129 standard deviations higher than the mathematics level of girls (0.029) (Table A3 in Appendix A). Again this is in line with the gender gap in mathematics found in other data, e.g., TIMSS grade 4 in Fig. 1.

Due to being constructed from categorical variables, the distribution of mathematics levels is trimodal: about half of the distribution is around the mean, and 25–25% are below or above the mean (Fig. 3). Measuring objective mathematics abilities well is key for our analysis, so we provide several robustness checks to our main results to show that measurement error does not drive our results. These robustness checks are detailed in Sect. 4.

#### Mathematics test scores

At age 12, study members also completed an Internet-based mathematics test. The scores of this test have been standardized to mean zero and standard deviation one, and follow a normal distribution (Figure Appendix A3 in Appendix A). Robustness tests using these scores are detailed in Section 4.

### SAMA along the levels of mathematics abilities

**Fig. 4:**
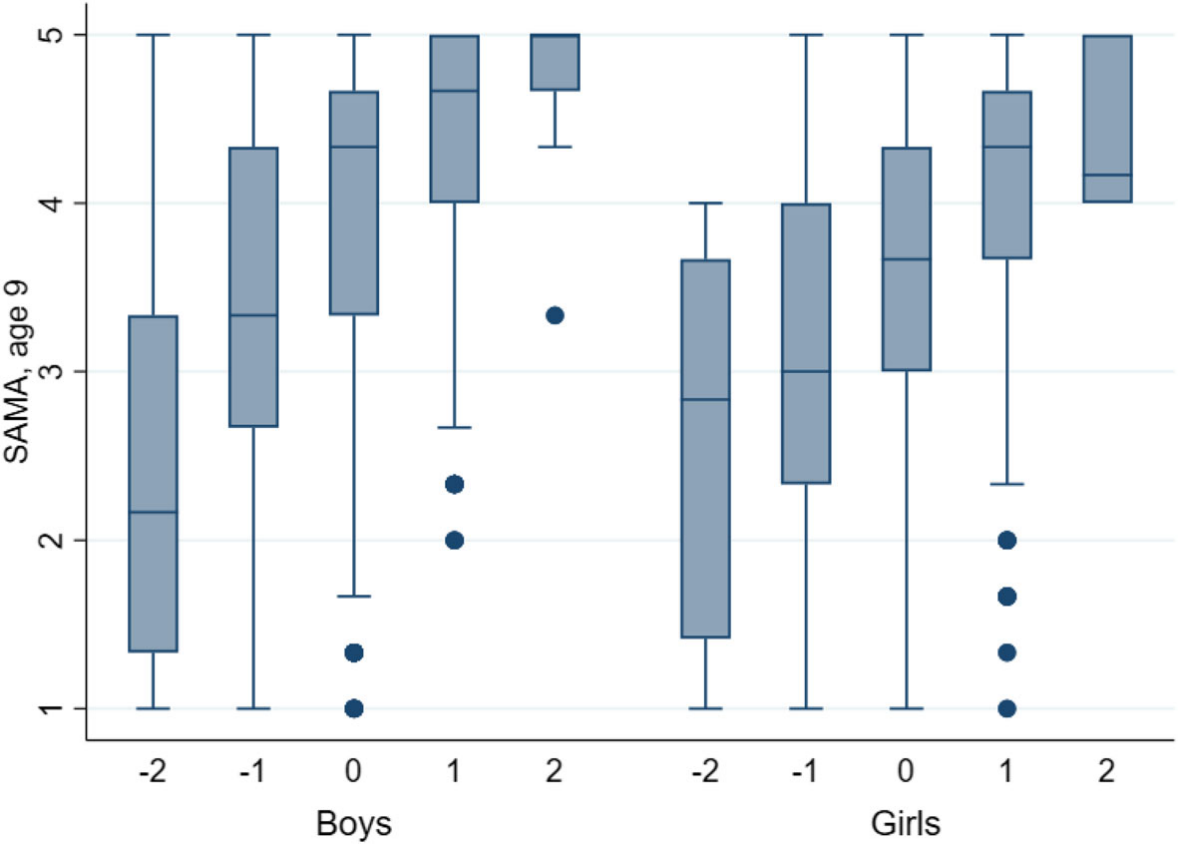
The distribution of self-assessed mathematics over standardized mathematics levels, age nine. *Notes:* *N* = 3877. The bottom of each box represents the 25th percentile, the center line represents the 50th percentile, and the top line represents the 75th percentile. The “whiskers” below and above the box represent the lower and upper adjacent values, defined as the 25th percentile minus 1.5 times the interquartile range and the 75th percentile plus 1.5 times the interquartile range. The dots represent outliers. Source: TEDS (Rimfeld et al. 2019)

Figure 4 shows the distribution of SAMA across the standardized measure of mathematics performance. At levels above average (greater than zero), the female distributions of SAMA display more variance and a lower mean than the male distributions, which indicates that even very high-achieving girls rate their mathematics ability lower than high-achieving boys.

### Control variables

In addition to objective skills in math, we control for a range of control variables in our models. Our main variable of interest is gender (a dummy variable for being female), which captures the gender gap. In TEDS there is no distinction between sex at birth and gender, so we use gender throughout this paper as it more accurately captures the experience of living as a man or woman (in this case boy or girl).

We also include measures of cognitive abilities since they are related to how accurately individuals self-assess their ability. Evidence from psychology tells us that lower-ability individuals have a more difficult time on the “meta-cognitive” task of self-assessment (i.e., the Dunning-Kruger effect) and therefore it is important to include measures of cognitive ability in any models where self-assessment is the outcome (Dunning et al. 2004). TEDS measures objective cognitive abilities via tests taken at various ages. In our analysis, we use cognitive ability measures from age nine in our main models, while also providing robustness checks using cognitive ability measures from age seven and 12. To accommodate the potentially heterogeneous gender gap in different types of cognitive skills, we use two separate cognitive skill indexes, one for verbal skills and one for non-verbal skills.

We also control for individual characteristics that might affect mathematics outcomes and mathematics self-assessment. This includes whether individual *i* is the elder twin (i.e., born first); whether individual *i* was heavier at birth than the co-twin; and birth weight in grams. Descriptive statistics of all variables are shown in Table A2 in Appendix A.

Finally, we include cohort fixed effects to account for the fact that the age nine wave of TEDS covers two school cohorts, born between 1994 and 1996. As consecutive school cohorts might differ from each other or might be exposed to different circumstances, we control for cohort-fixed effects in all models.

### Potential channels

We use the following variables to test the three potential channels outlined in Sect. 2.

#### Sibling peer effects

Having a boy co-twin (as opposed to having a girl co-twin) could affect both girls and boys through increased in-utero testosterone exposure, as well as create a different environment in the family. We capture this by including a dummy variable for having a boy co-twin. Unfortunately, we cannot fully disentangle the biological from the environmental explanation via this dummy variable.

We also want to account for having a brother, since it may be about having any brother not necessarily a twin brother. In some models, we therefore include a dummy variable for “having a brother” to capture the experience of growing up with brothers apart from one’s co-twin. Note that most siblings are older than the twins in the data since once parents have twins they tend to end their fertility. Only 1% of the sample has a younger brother, while among those who have brothers (32%), only 2.7% have a younger brother. This means that using whether the individual has an older brother (as opposed to just a brother) would lead to similar results.

To dig deeper in the peer effects channel, we look at the role of the co-twin’s mathematics level and their self-assessed ability. We include the co-twin’s mathematics level to understand how self-assessments are related to the ability level of one’s twin. We also include co-twin’s self-assessments since they may be related to individual self-perceptions. This includes their SAMA, as well as their self-assessed English and physical abilities. Self-assessed English and physical abilities are captured similarly to SAMA. For English, the survey asks three questions: How good do you think you are at reading, writing, and spelling? All potential answers are coded using a Likert scale from 1 to 5, and the average of the three questions is provided in the data. For physical abilities, the survey again asks three questions: How good do you think you are at playing team games, races and competitions, and physical education classes? All potential answers are coded using a Likert scale from 1 to 5, and the average of the three questions is provided in the data.

#### Transmission of adult stereotypes

To probe the channel of adult stereotypes, we include parental and teachers’ assessments of the mathematics abilities of the twins. The questions are the same as for the SAMA measure (How good do you think your child is at: solving number and money problems, doing Maths in their head, and multiplying and dividing). The adults also answer using five ordinal answers to each: very good; quite good; doing OK; not so good; not good at all, coded using a Likert scale from one (worst) to five (best). The average of the three responses is provided in the data.

To dig further into this channel, we construct a measure of gender-stereotypical parental assessment. This allows us to understand if adult stereotypes are more binding in more traditional households. To do this, we construct a binary variable that captures whether parents’ assessment of their children’s mathematics abilities is stereotypically gender-biased. This variable takes the value one if they either: overestimate their son in math or underestimate their daughter in math. The variable is child-specific and may vary within twins/families.

We determine over- and underestimation by comparing the mathematics levels of the twins and the parental assessments of children. First, we model the assessment category given by parents using a multinomial logit model, where we condition on objective mathematics levels as well as verbal and non-verbal cognitive skills measured at age nine. Then, we compare the category given by parents to the category predicted by the model to determine whether parents over- or underestimate their children’s mathematics skills.

In our main results, we use the terciles of parental assessments as the outcome variable in these models due to the distribution of the parental assessments (hence we model three categories). We also provide a robustness check where instead of terciles, we use the parental assessment level on a 1–5 scale (taking the integer of the parental assessment values, that are the average levels given in response to the three questions as for SAMA) which results in a five-category model. The two methods lead to very similar results.

The gender gap in parental assessments is presented in Table A3 in Appendix A. Boys are more likely to be overestimated while girls are more likely to be underestimated in mathematics. Overall, 26% of young people received a stereotypically gender-biased assessment from their parents (Table A2 in Appendix A).

#### Comparative advantage of girls in English compared to mathematics

We test whether those with higher abilities in English have lower SAMA and whether such relationship is heterogeneous by gender. English abilities are measured similarly to mathematics abilities using National Curriculum levels from 1 to 5, given by the teachers.

We also test whether SAMA is related to self-assessed English abilities (as a proxy for confidence in general) by including this variable in the model.

## Empirical methods

Our goal is to robustly estimate the gender gap in SAMA, controlling for as much unobserved heterogeneity as possible. We begin using linear regression models. First, we estimate the following model:

1 $$\begin{aligned} SAMA_{i,j}=\alpha + \beta _{OLS}female_{i,j} + X_{i,j}\delta + u_{i,j} \end{aligned}$$

where

*j*: represents the twin pair
*i*: represents the individual within a twin pair
$$female_{i,j}$$
is our gender dummy and captures whether individual *i* is a girl
$$X_{i,j}$$
is a matrix of control variables discussed in the previous section
$$u_{i,j}$$
is the usual error term, robust and clustered by twins.

In this model, $\beta _{OLS}$, the estimated parameter on our variable for female, captures the gender gap in the outcome variable, conditional on $X_{i,j}$.

Our preferred empirical model, however, also controls for twin-pair fixed effects (FE). Whenever possible, i.e., when we do not want to control for individual characteristics that are constant within twin pairs, we use twin-pair FE models. These models identify the gender gap within boy-girl twin pairs and allow us to account for the shared genetic and home environment common to the twin pair. To do this, we estimate variations of the following model:

2 $$\begin{aligned} SAMA_{i,j}=\alpha + \beta _{FE}female_{i,j} + X_{i,j}\delta + \nu _j+u_{i,j} \end{aligned}$$

where $\nu _j$ is the twin-pair fixed effect, and all other variables are as previously outlined. $\beta _{FE}$ captures the within-twin-pair gender gap in the outcome variable.

We estimate our models additively, beginning with the simple regression of SAMA on the female dummy in Model 1. This is extended to include mathematics performance at age nine in Model 2. This allows us to examine whether boys are more confident in their mathematics ability as compared to girls who have the same level of performance. In Model 3, we introduce additional cognitive ability controls as well as individual demographic characteristics, which may drive some of the gender gap in SAMA. In Model 4, we introduce twin-pair fixed effects. This allows us to control for unobserved heterogeneity common to the twin pair, e.g., shared genes and family environment.

We provide the following robustness tests to our main models on SAMA. First, we re-estimate our main models treating the mathematics level variable as categorical. We do this because the mathematics-level variables were constructed from three categorical variables and about 50% of observations are around the mean.

Second, we address issues of measurement error. The measurement of objective mathematics skills is key to estimating the gender gap in SAMA over and above objective mathematics performance. Furthermore, applying FE models might exacerbate any measurement error issues (Collischon and Eberl 2020). Thus, we aim to reduce measurement error in mathematics levels in six ways. First, we also control for mathematics levels and verbal and non-verbal cognitive skills from age seven (on the overlap sample of those who participated in age seven and nine data collection). Second, as participants completed a mathematics test at age 12, we repeat the estimation on the age 12 sample (measuring SAMA at age 12) and also control for age 12 test scores on top of mathematics levels. Third, exploiting the overlap sample of the age nine and 12 data collections, we re-estimate our main model on age 12 SAMA while controlling for both age nine and age 12 mathematics levels and age 12 mathematics test scores as well. Fourth, we repeat the previous exercise by controlling for age seven, nine, and 12 level and test scores variables at the same time. Note that the overlap samples have fewer observations. Our last two methods are two instrumental variable approaches. First, following Ladd and Walsh (2002), we instrument age nine math levels by age seven math levels. Second, we follow the ORIV approach of Gillen et al. (2019), which uses both age nine math levels to instrument age seven math levels and age seven math levels to instrument age nine math levels at the same time. All these methods lead to similar results.

In our third robustness test, we investigate whether the gender gap varies along the distribution of SAMA. We treat SAMA as a categorical variable (as opposed to continuous) and estimate a multinomial logistic model.

Lastly, we re-estimate our main results on a sub-sample that only contains dizygotic twins. Boy-girl twins are dizygotic by nature, so we test what happens when we exclude monozygotic twins from the analytical sample.

### Exploring the channels

We explore the role of the potential channels outlined in Sect. 2 by extending the main model with variables accounting for the three channels as well as their interaction with the female dummy to explore heterogeneous effects.

First, we estimate a series of models to account for sibling peer effects. These models allow us to examine the role of siblings (reference point) in the gender gap in SAMA. We do this by including a dummy variable for whether an individual’s co-twin is a boy to the model without twin fixed effects. We then introduce an interaction term for whether the individual is a girl and their co-twin is a boy. In a separate model, we replace having a male co-twin with having a brother (who is not their twin pair) to test whether the same relationship occurs as for having a male co-twin. Finally, we estimate this last model separately for boy-girl and boy-boy/girl-girl twins to investigate the consequences of having a brother separately for girls who have or do not have a male co-twin.

We further probe the peer effects explanation by including further characteristics of the twin beyond their gender, namely their SAMA and mathematics levels. This allows us to delve further into the reference point hypothesis and explore whether their co-twin’s ability and SAMA might discourage girls and explain part of the gender gap. Lastly, we repeat this last exercise for two further facets of self-assessment: self-assessed English and physical abilities.

Second, we extend the main model to account for adult stereotypes. We introduce the measures of parental and teacher mathematics assessments. Then we introduce the variable capturing whether one received a stereotypically gender-biased parental assessment, as well as the interaction term of this variable with female. Again, we estimate linear models with OLS and twin-pair FE models.

Lastly, we extend the main model with objective measures of English ability. Then, we add self-assessed English ability, as well as the interactions of both variables with female. We estimate these three new models using OLS and twin-pair FE models.

## Results

### Main results

Table 1 presents the main results obtained from estimating Eq. 1 on the age nine sample. In all models, the coefficient of interest is on the female dummy, indicating the difference between boys and girls. Model 1 reveals a large and statistically significant raw gender gap in SAMA of $-$0.38 standard deviations. Girls rate their own mathematics ability nearly 40% of a standard deviation lower than boys. In Model 2, this is reduced by the inclusion of mathematics ability by five percentage points (13%), but still large ($-$0.33 SD) and statistically significant. This result indicates that a girl with the same mathematics skills as her male peer still rates her mathematics ability one-third of a standard deviation lower on average. This is large when we consider that the gender gap in actual mathematics performance is closer to 10% of a standard deviation on average.

**Table 1:** The gender gap in mathematics self-assessment (SAMA), age nine

	Model 1	Model 2	Model 3	Model 3	Model 4
				OS subsample	
	(1)	(2)	(3)	(4)	(5)
Female	$-$0.376***	$-$0.328***	$-$0.324***	$-$0.449***	$-$0.447***
	(0.034)	(0.032)	(0.032)	(0.051)	(0.051)
Math level, age 9		0.372***	0.327***	0.319***	0.359***
		(0.016)	(0.019)	(0.030)	(0.032)
Verbal abilities, age 9			0.054***	$-$0.030	0.082**
			(0.018)	(0.030)	(0.035)
Non-verbal abilities, age 9			0.061***	0.110***	0.131***
			(0.020)	(0.034)	(0.033)
Elder twin			0.038	0.062	0.034
			(0.026)	(0.051)	(0.027)
Heavier twin at birth			0.042	0.051	0.039
			(0.028)	(0.055)	(0.043)
Birth weight, grams			0.000	$-$0.000	0.000
			(0.000)	(0.000)	(0.000)
Constant	0.182***	0.118***	$-$0.064	0.251*	0.033
	(0.035)	(0.032)	(0.089)	(0.151)	(0.236)
Observations	3877	3877	3877	1186	3877
R-squared	0.036	0.165	0.174	0.195	0.164
Twin FE	No	No	No	No	Yes
Cohort FE	Yes	Yes	Yes	Yes	No

*Notes:* Source: TEDS (Rimfeld et al. 2019). Robust standard errors clustered by twin pairs in parentheses. *p < 0.1; **p < 0.05; ***p < 0.01

In Model 3, we exploit the rich nature of the TEDS data and include a range of control variables for cognitive ability as well as individual characteristics. These do very little to reduce the gender gap in SAMA ($-$0.32 SD; six percentage points or 16% smaller than the raw gap). This indicates that differences in cognitive ability cannot explain the gender gap in self-perceptions.

In Model 4, we restrict the sample to boy-girl twins while in Model 5, we introduce twin-pair fixed effects on top of the aforementioned control variables. This means we estimate our gender gap within boy-girl twin pairs as outlined in Eq. 2. Interestingly, the gender gap increases in magnitude to $-$0.45 SD.^2^

In Table O6 in the Online Appendix, we repeat the same estimations by adding the interaction terms of female and all control variables to the model to see any potential differential effects. Returns to mathematics levels in terms of SAMA do not differ between men and women (Model 2). In the OLS model (Model 3), none of the interaction terms are statistically significant or meaningful in magnitude, while in the FE model (Model 4), the interaction term of female and verbal skills is significant and negative. Thus, within boy-girl twin pairs, girls’ SAMA is negatively correlated with their verbal abilities.

In Tables O7 and O8 in the Online Appendix we investigate further whether the gender gap in SAMA differs along the ability distribution in a non-linear fashion by constructing categorical variables from math levels and non-verbal skills. As Fig. 3 shows, the distribution of math levels is trimodal: most people (64%) are around the middle, while some people are below (15%) or above (21%) the middle. Thus, based on this variable, we create a categorical variable that is 0 for the middle category, 1 for those above, and 2 for those below the middle category. Introducing the interaction term of this variable with female results in insignificant interaction terms; i.e., the gender gap in SAMA is neither lower nor higher among those with lower or higher math grades. Similarly to this, we also look at whether one‘s nonverbal cognitive skills are above (high ability group) or below (low ability group) the median and interact this variable with gender. Again, we find that the estimated interaction coefficients are not statistically significant, i.e., the gender gap does not differ between those with high vs low ability at age 9. These results underline our previous findings that actual abilities only play a limited role in the gender gap in math self-assessment. This is true whether we use our main specification as Models 3 and 4 in Table 1 (Table O7 in the Online Appendix), or we exclude those control variables that capture abilities (math levels, verbal and non-verbal cognitive skills (Table O7 in the Online Appendix).

We provide the following robustness checks to support our results on the contribution of objective mathematics abilities to the gender gap in SAMA in Appendix B. First, as the distribution of mathematics levels is trimodal (Fig. 3), we control for mathematics levels as a categorical variable in Column 1 and Column 4 of Table B1. This does not change the results.

Second, we try to reduce any potential measurement error in mathematics levels at age nine in various ways. We control for mathematics levels and cognitive skills at age seven (Columns 3 and 6 of Table B1), as well as using two types of IV strategies (Tables B2 and B3). While the overlap sample of the age seven and age nine data is somewhat smaller than our main analytical sample, the conditional gender gap is similar, and not different from the earlier estimates.

We also repeat the estimation using age 12 SAMA as the dependent variable in Table B5. The age 12 raw gender gap in SAMA is similar in magnitude to the age nine gap (note that most of the age 12 sample covers different individuals as compared to the age nine sample, the overlap of the two is only 570 individuals), $-$0.39 standard deviation (Model 1). Controlling for age 12 mathematics levels decreases the gap by 14.5 to $-$0.34% (Model 2). Once we also control for age 12 test scores and age 12 cognitive skills, the gap decreases further to $-$0.299 (Model 3). Thus, all age 12 mathematics and cognitive skill measures explain 24.1% of the gender gap in SAMA at age 12.

When we restrict the sample to those with both age nine and age 12 data and control for age 12 and age nine mathematics and cognitive skill measures as well, the gender gap in SAMA is still 0.34 standard deviations (Model 4). When we restrict the sample further to those with age seven, age nine, and age 12 data and control for all available measures from the three ages, the gap is still 0.29 standard deviations (Model 5). Repeating the same exercise in twin FE models yields similar results (Table B6), as well as restricting the sample to dizygotic twins (Table B4). This highlights the stability of the results across samples and ages.

Next, we treat SAMA as if it was categorical in a multinomial logit model and show that the gender gap is the largest at the top of the mathematics skills’ distribution (Table B7).

Lastly, as mentioned in Sect. 3, we re-estimate Table 1 using three different sets of weights to take the selection into the analytical sample into account in the Online Appendix. Table O3 shows that our results stay similar, suggesting that the selection to the sample is not a serious concern in this case.

**Table 2:** The role of sibling composition in the gender gap in SAMA

				Model 1	Model 3	Model 1	Model 3
	Model 1	Model 2	Model 3	OS twins	OS twins	SS twins	SS twins
	(1)	(2)	(3)	(4)	(5)	(6)	(7)
Female	$-$0.354***	$-$0.362***	$-$0.297***	$-$0.449***	$-$0.436***	$-$0.270***	$-$0.239***
	(0.032)	(0.045)	(0.038)	(0.051)	(0.061)	(0.040)	(0.048)
Has a male twin (MT)	$-$0.078**	$-$0.086*					
	(0.032)	(0.045)					
Female*MT		0.016					
		(0.067)					
Has brother			0.042		0.068		0.034
			(0.047)		(0.077)		(0.059)
Female*has brother			$-$0.084		$-$0.046		$-$0.094
			(0.068)		(0.109)		(0.085)
Constant	$-$0.014	$-$0.007	$-$0.076	0.251*	0.256*	$-$0.193*	$-$0.207*
	(0.091)	(0.094)	(0.089)	(0.151)	(0.152)	(0.108)	(0.109)
Observations	3877	3877	3877	1186	1186	2691	2691
R-squared	0.175	0.175	0.174	0.195	0.196	0.171	0.171
Twin FE	No	No	No	No	No	No	No
Cohort FE	Yes	Yes	Yes	Yes	Yes	Yes	Yes
Sample	Total	Total	Total	OS twins	OS twins	SS twins	SS twins

*Notes:* Source: TEDS (Rimfeld et al. 2019). Further control variables: mathematics level at age nine, verbal and non-verbal cognitive skills at age nine, elder twin, heavier twin, and birth weight. “SS twins” refers to boy-boy or girl-girl twins. “OS twins” refers to boy-girl twins. Robust standard errors clustered within twin pairs in parentheses. *p < 0.1; **p < 0.05; ***p < 0.01

### Sibling peer effects

We now turn our attention to potential peer effects explanations for the gender gap in SAMA. Table 2 confirms our earlier result that having a male co-twin reduces SAMA (Model 1), as we saw before that the gender gap in SAMA is larger among boy-girl twins. We do not find evidence for a gender-specific relationship because the interaction term of having a male co-twin with female is not significantly different from zero (Model 2).

Our setup does not allow us to test whether the negative effect of having a male co-twin is biological (i.e., stems from in-utero testosterone exposure) or is the result of the different environment into which these young people were born (as opposed to having a same-sex twin). We can however test what happens if we look at the relationship between SAMA and having a brother (who is not one’s male twin) in general. Note that as mentioned earlier, most siblings in the data are older than the twins,^3^ so this exercise is nearly the same as looking at older brothers. In Model 3, we control for having a brother as opposed to having a male twin, but we do not find a statistically significant relationship neither for boys nor for girls. However, when we test whether the estimated coefficients on having a male twin in Model 2 vs. having a brother in Model 3 differ from each other in a statistical sense, we find gender-specific results. For boys, testing whether the coefficients on having a male twin from Model 2 ($-$0.086) and having a brother from Model 3 (0.042) are equal yields a difference of $-$0.128 with a chi-squared test p-value of 0.0435. Thus, for boys, SAMA has a larger negative correlation with having a male twin compared to having a brother. For girls, we tested the equality of “has a male twin” + “female * has a male twin” from Model 2 vs. “has brother” + “female * has brother” from Model 3. This yields a difference of ($-$0.0861+0.0157)-(0.0417$-$0.0836)=$-$0.0285 with a chi-squared test *p*-value of 0.6917. Thus, for girls, the correlation between SAMA and having a male twin vs. having a brother is not statistically significantly different from each other.

In Columns 4 and 5, we restrict the sample to boy-girl twin pairs to look separately at girls with male twins. Repeating Model 3 on this sub-sample (Column 5) does not show a relationship between SAMA and having a brother (on top of one’s male co-twin).

Lastly, in Columns 6 and 7, we look at the sub-sample of boy-boy and girl-girl pairs (pooling all twin pairs together). None of the girls in this sub-sample have a male co-twin. The gender gap among twins of the same gender is smaller than the average, 0.27 standard deviation (Column 6), which is consistent with our previous findings showing a larger-than-average gap for boy-girl twins. Controlling for having a brother and its interaction term with female in Column 7 shows that the gender gap in SAMA is slightly smaller among those who do not have brothers. Although the interaction term of female and having a brother is not statistically significant, it is modest, $-$0.09 SD. These results are not robust enough to draw a strong conclusion about the role of biological versus environmental factors in the negative association between SAMA and the gender composition of siblings. However, as mentioned above, for girls, the relationship between having a male twin versus a non-twin brother and SAMA is similar. For boys, only having a male twin is negatively correlated with SAMA, having a non-twin brother is not.

In Table 3, we look at the role of the SAMA of co-twins. On average, own SAMA is positively correlated with co-twin SAMA (Model 1), and this relationship is not different for boys and girls (Model 3). Furthermore, the SAMA of one’s co-twin does not change the previously found negative relationship between having a male co-twin and own SAMA (Model 3). Introducing the triple interaction term of female, having a male co-twin, and co-twin SAMA,^4^ however, reveals that male co-twin SAMA matters differently for boys and girls (Model 4).

For a simpler interpretation, we re-estimate Model 4 separately for boys and girls in Columns 5 and 6. For boys (Column 5), SAMA is positively correlated with their male co-twin’s SAMA (0.158), while the SAMA of their female co-twin is smaller in magnitude (0.033) and not statistically significant. For girls, it is also true that their SAMA is positively correlated with their girl co-twin’s SAMA (0.245), however, their SAMA is negatively correlated with their male co-twin’s SAMA. In other words, among boy-boy and girl-girl twins, high self-assessment is mutually beneficial. Among boy-girl twins, female SAMA is negatively correlated with male SAMA.^5^ Note that this phenomenon does not occur for objective mathematics abilities: the objective mathematics levels of male co-twins do not matter for the gender gap in SAMA (Table O16 in the Online Appendix).

Interestingly, if we repeat the same exercise looking at the gender gap in self-assessed English or physical abilities, we find the same pattern. The confidence of a male co-twin is related to the confidence of boys but not to girls’ in English (Table O18 in the Online Appendix) and physical abilities (Table O19 in the Online Appendix).

**Table 3:** The role of co-twin (CT) SAMA

					Model 4	Model 4
	Model 1	Model 2	Model 3	Model 4	boys	girls
	(1)	(2)	(3)	(4)	(5)	(6)
Female	$-$0.326***	$-$0.326***	$-$0.383***	$-$0.342***		
	(0.030)	(0.031)	(0.035)	(0.033)		
Has a male twin (MT)			$-$0.151***	$-$0.127***	$-$0.127***	$-$0.122***
			(0.036)	(0.033)	(0.044)	(0.047)
SAMA of CT, age 9, std	0.153***	0.127***	0.181***	0.034	0.033	0.245***
	(0.023)	(0.029)	(0.028)	(0.036)	(0.036)	(0.036)
MT*SAMA of CT			$-$0.034	0.162***	0.158***	$-$0.206***
			(0.039)	(0.054)	(0.055)	(0.057)
Female*SAMA of CT		0.048		0.207***		
		(0.037)		(0.050)		
Female*MT*SAMA of CT				$-$0.365***		
				(0.094)		
Constant	$-$0.047	$-$0.046	0.047	0.025	$-$0.019	$-$0.272**
	(0.081)	(0.081)	(0.085)	(0.083)	(0.114)	(0.109)
Observations	3722	3722	3722	3722	1707	2015
R-squared	0.196	0.197	0.201	0.208	0.205	0.158
Twin FE	No	No	No	No	No	No
Cohort FE	Yes	Yes	Yes	Yes	Yes	Yes

*Notes:* Source: TEDS (Rimfeld et al. 2019). Further control variables: mathematics level at age nine, verbal and non-verbal cognitive skills at age nine, elder twin, heavier twin, and birth weight. CT refers to co-twins. Robust standard errors clustered within twin pairs in parentheses. *p < 0.1; **p < 0.05; ***p < 0.01

Lastly, for easier interpretation, we re-estimate Table 3 using a binary variable capturing very high co-twin SAMA instead of the original continuous variable (Table O17 in the Online Appendix). We create a binary variable for having a “confident twin” that equals one if the co-twin’s SAMA falls in the top 20% of the distribution and zero otherwise. This exercise shows that indeed, among boys, having a confident male twin is related to having higher SAMA ($-$0.158+0.127+0.234=0.203), but this is not the case among girls (0.002+0.403$-$0.485=$-$0.08). This result is in line with some of the previously discussed literature on men versus women’s reaction to competition if we think that having a very confident closest peer might create a more competitive environment.

### The transmission of gender stereotypes

The transmission of gender stereotypes from adults to children may be an important driver of the gender gap in SAMA. Unfortunately, we are unable to identify the causal effects of parental assessments on their children’s assessments, as these two can mutually enforce each other and thus are endogenous. However, we explore whether there is a gender gap in how parents and teachers assess the mathematics ability of boys and girls. After finding gender differences in these assessments, we then control for them in our main model.

In Tables O12 and O13 in the Online Appendix, we estimate the same models as in Table 1, but now the outcome variable is either parent or teacher assessment of the twins’ mathematics ability instead of SAMA. The main results are broadly similar. Parents assess girls’ mathematics ability lower than boys’ even once we account for their actual mathematics performance (Model 2, approximately $-$0.2 SD). Interestingly, the difference is even more pronounced between boys and girls within the same twin pair (Model 5). Here parents assess their daughters’ mathematics ability $-$0.42 SD lower than their male twins.

The gender gap in teachers’ assessment of boys’ and girls’ mathematics ability is similar in magnitude to parents’ assessment in raw terms ($-$0.2 SD), but halves once we account for actual mathematics ability, i.e., mathematics levels given by the same teachers ($-$0.12 SD).^6^ Teachers should have more accurate knowledge about the children’s actual mathematics ability, so this is unsurprising. Including twin fixed effects in the model does not change the estimated coefficient significantly. Next, we explore the inclusion of parent and teacher assessments as a potential channel by including them in our main models of SAMA.

**Table 4:** The role of parental and teachers assessments in the gender gap in SAMA

	Model 1	Model 2	Model 3	Model 4	Model 5	Model 6
	(1)	(2)	(3)	(4)	(5)	(6)
Female	$-$0.227***	$-$0.079	$-$0.288***	$-$0.099	$-$0.217***	$-$0.004
	(0.029)	(0.141)	(0.031)	(0.132)	(0.029)	(0.147)
Parental assessment of Math	0.498***	0.518***			0.465***	0.463***
	(0.021)	(0.028)			(0.022)	(0.031)
Female*parental assessment		$-$0.037				0.005
		(0.033)				(0.040)
Teachers’ assessment of Math			0.355***	0.384***	0.161***	0.197***
			(0.029)	(0.033)	(0.028)	(0.033)
Female*teachers’ assessment				$-$0.056		$-$0.069*
				(0.036)		(0.041)
Constant	$-$1.908***	$-$1.989***	$-$1.237***	$-$1.331***	$-$2.321***	$-$2.429***
	(0.112)	(0.134)	(0.128)	(0.139)	(0.131)	(0.148)
Observations	3877	3877	3877	3877	3877	3877
R-squared	0.308	0.309	0.207	0.207	0.315	0.315
Twin FE	No	No	No	No	No	No
Cohort FE	Yes	Yes	Yes	Yes	Yes	Yes

*Notes:* Source: TEDS (Rimfeld et al. 2019). Further control variables: mathematics level at age nine, verbal and non-verbal cognitive skills at age nine, elder twin, heavier twin, and birth weight. Robust standard errors clustered within twin pairs in parentheses. *p < 0.1; **p < 0.05; ***p < 0.01

The models in Table 4 highlight the importance of parental perceptions in explaining the gender gap. Model 1 shows a decrease of approximately 30% when we introduce parental assessments of their children’s mathematics ability (from $-$0.32 SD in Table 1 to $-$0.23 SD). This is larger than the share of the gender gap that was explained by actual mathematics performance. The coefficient on the interaction term of female with parental assessment in Model 2 is not significant. This means that parental assessment in general does not have a different correlation with the SAMA of boys and girls.

Compared to the main model (Model 3 in Table 1), the gap is also reduced somewhat when we control for teacher assessments in Column 3, but not by as much. Again, the interaction term of teachers’ assessments and female is not significant (Column 4). Introducing both sets of adult assessments in Model 5 reduces the gap slightly more, but it seems as though most of the reduction is led by parental assessments. Introducing the interaction terms of parental and teachers’ assessments with gender reveals that conditional on parental assessment, girls’ SAMA is negatively correlated with teachers’ assessments (Model 6). The same models estimated with twin-pair/family FEs are shown in Table O14 in the Online Appendix.

**Table 5:** The role of stereotypically gender-biased parental assessments in the gender gap in SAMA

	Model 1	Model 2	Model 3	Model 4	Model 5	Model 6
	(1)	(2)	(3)	(4)	(5)	(6)
Female	$-$0.324***	$-$0.324***	$-$0.032	$-$0.447***	$-$0.447***	$-$0.146**
	(0.032)	(0.032)	(0.037)	(0.051)	(0.051)	(0.061)
Stereotypically assessed person		0.002	0.576***		0.027	0.512***
		(0.034)	(0.043)		(0.051)	(0.076)
Female*stereotypically assessed			$-$1.093***			$-$0.961***
			(0.067)			(0.116)
Constant	$-$0.064	$-$0.065	$-$0.139	0.033	0.027	$-$0.090
	(0.089)	(0.089)	(0.085)	(0.236)	(0.237)	(0.230)
Observations	3877	3877	3877	3877	3877	3877
R-squared	0.174	0.174	0.227	0.164	0.164	0.199
Twin FE	No	No	No	Yes	Yes	Yes
Cohort FE	Yes	Yes	Yes	No	No	No

*Notes:* Source: TEDS (Rimfeld et al. 2019). Further control variables: mathematics levels at age nine, verbal and non-verbal cognitive skills at age nine, elder twin, heavier twin, and birth weight. The measure of parental stereotypical assessment was created the following way. First, a multinomial logit model of the form $f(k,i)=beta_k*x_i$ is estimated, where $beta_k$ is a set of regression coefficients associated with the *k* terciles of parental assessments, $k={1,2,3}$, and $x_i$ is the set of three explanatory variables: mathematics levels and verbal and non-verbal cognitive skills at age nine. Then, predicted categories of parental assessment are fitted by the model and they are compared to the observed parental assessment of individuals. An individual is over(under)estimated if their observed parental assessment category is higher(lower) than their predicted category. Robust standard errors clustered within twin pairs in parentheses. *p < 0.1; **p < 0.05; ***p < 0.01

We introduce our measure of stereotypically gender-biased parental assessment as explained above^7^ in Table 5. Compared to the gender gap in our main model ($-$0.32 SD, Model 1 in Table 1), the gap does not change when we introduce the measure in Model 2 ($-$0.32 SD). However, when we also introduce its interaction term with gender (Model 3), the gender gap among those who did not receive a stereotypically biased parental assessment becomes small and insignificant ($-$0.03 SD). This shows that the average difference between the SAMA of non-underestimated girls and non-overestimated boys is statistically negligible. The coefficient on the stereotypical assessment measure is positive and significant (0.58 SD), indicating that the average SAMA of overestimated boys is larger than that of non-overestimated boys^8^.

Lastly, the estimated coefficient on the interaction term is large and highly significant ($-$1.1 SD). This suggests that the average SAMA of underestimated girls is more than one standard deviation lower than the SAMA of overestimated boys. Results are similar in the twin FE setup (Models 4–6), when we use our alternative measure of parental stereotypical assessments (Table B12 in Appendix B), and also when we apply our IV strategies for measurement error (Tables B13 and B13 in Appendix B).

The fact that stereotypical parental assessments are associated with SAMA raises the question of how they might be related to within-twin peer effects (that we explore in Table 3). Table O22 in the Online Appendix investigates this question. We split the samples’ boys and girls to sub-samples of stereotypically assessed individuals (i.e., overestimated boys and underestimated girls) and not-stereotypically assessed individuals (not overestimated boys and not underestimated girls), resulting in four sub-samples. Interestingly, among girls, it does not matter whether they are stereotypically assessed by their parents or not: the large negative correlation between their SAMA and the SAMA of their male co-twin is the same in the two female sub-samples (Columns (3) and (4)). Among boys, however, the positive correlation between their own SAMA and the SAMA of their co-twin is only there among overestimated boys. This suggests that the relationship we find for girls is probably more society-driven, while the association for boys is more family-driven.

Re-weighting these models with the three types of weights introduced above also leads to similar conclusions (Table O4 in the Online Appendix). These results suggest that gender-biased parental assessments play a large role in the gender gap in SAMA.^9^

### The role of girls’ comparative advantage in English

Table 6 investigates the role of girls’ comparative advantage in English in the gender gap in SAMA. In our sample, girls are 0.256–0.353 SD better in English than boys (Table O11 in the Online Appendix). Extending our main models, Model 3 and Model 4 in Table 1, by controlling for English ability (levels) slightly decreases the gap by about 5–10% (from 0.32 to 0.28 SD in the OLS model and from 0.45 to 0.43 SD in the FE model). The coefficients on English levels are statistically significant and negative: those with better English skills have lower confidence in their mathematics skills, conditional on their mathematics abilities.

When self-assessed English ability is also added to the model in Columns 2 and 5, the gender gap bounces back to the earlier levels. SAMA is positively correlated with English self-assessment in all models. The interaction terms with the gender dummy are negative with English levels and positive with English self-assessment, but the former is only significant in the FE model (Column 6) while the latter is only significant in the OLS model (Column 3). Thus, in general, the positive correlation between confidence in English and confidence in mathematics is about 30% larger for girls than for boys. Among boy-girl twins, however, English levels seem to matter more for girls: English levels decrease girls’ SAMA two times as much as boys’ confidence in math. This would lend some support for a comparative advantage story in boy-girl twin pairs where the boy may already identify as the “mathematics person.”

**Table 6:** The role of girls’ comparative advantage in English in the gender gap in SAMA

	Model 1	Model 2	Model 2	Model 4	Model 5	Model 6
	(1)	(2)	(3)	(4)	(5)	(6)
Female	$-$0.283***	$-$0.318***	$-$0.734***	$-$0.425***	$-$0.462***	$-$0.798***
	(0.033)	(0.031)	(0.211)	(0.053)	(0.052)	(0.294)
English level, age 9	$-$0.116***	$-$0.214***	$-$0.186***	$-$0.067	$-$0.129***	$-$0.083*
	(0.024)	(0.023)	(0.028)	(0.043)	(0.043)	(0.049)
Perceived English, age 9		0.387***	0.334***		0.229***	0.185***
		(0.026)	(0.035)		(0.036)	(0.050)
Female*English level			$-$0.050			$-$0.089*
			(0.033)			(0.052)
Female*Self-assessed English			0.102**			0.085
			(0.050)			(0.069)
Constant	$-$0.087	$-$1.664***	$-$1.445***	0.011	$-$0.897***	$-$0.739**
	(0.089)	(0.134)	(0.165)	(0.236)	(0.272)	(0.307)
Observations	3877	3877	3877	3877	3877	3877
R-squared	0.179	0.242	0.243	0.165	0.188	0.189
Twin FE	No	No	No	Yes	Yes	Yes
Cohort FE	Yes	Yes	Yes	No	No	No

*Notes:* Source: TEDS (Rimfeld et al. 2019). Further control variables: mathematics level at age nine, verbal and non-verbal cognitive skills at age nine, elder twin, heavier twin, and birth weight. Robust standard errors clustered within twin pairs in parentheses. *p < 0.1; **p < 0.05; ***p < 0.01

## Discussion

This paper examined the gender gap in self-assessed mathematics ability using rich data on twins born in the UK. Despite a range of literature on the gender gap in mathematics performance and STEM attainment more broadly, literature exploring the gender gap in the self-assessment of mathematics ability is limited. This is important since self-assessments in mathematics ability may translate into decisions to pursue STEM fields in higher education and the labor market. We set out to fill this gap and examine why boys are more likely to rate their mathematics ability higher than girls, even when their ability is the same.

We find that the gender gap in SAMA is about three times as large as the gender gap in objective mathematics ability. This is a stark result and highlights the importance of addressing gender gaps in self-perceptions. Objective skills can only explain 14–26% of the gender gap in SAMA, which means there is a high degree of mis-perception. The gender gap in SAMA is even larger among boy-girl twins than among non-related boys and girls. We probe these results further and explore three potential channels: sibling peer effects, the transmission of gendered stereotypes from adults to children, and girls’ comparative advantage in English.

Our results lend support for the channels of sibling peer effects and adult stereotypes. In terms of twin peer effects, we find that the SAMA of boys is positively correlated with the SAMA of a male twin, but this positive correlation is not present for girls. This supports the idea that within families, there might be a narrative of who is the “mathematics person” and who is not. Once this role has been taken (by the boy), it is difficult for the girl to view herself as a “mathematics person” as well. While the SAMA of one’s co-twin is undoubtedly endogenous, these results might highlight the role of environment in terms of growing up with a male sibling as one’s most direct point of comparison. Psychologists point to social comparison theory (Festinger 1954) and contrast effects (Morse and Gergen 1970) to describe how individuals shape their self-perceptions based on others, which falls under the umbrella of peer effects in the economics literature (Sacerdote 2011).

Surprisingly, the objective mathematics ability of the co-twin does not matter for either boys or girls, only their self-assessment. This again points to the importance of stereotypes and gender norms pervading sibling interactions as opposed to actual ability. While the mechanism behind these peer effects is still not entirely clear, but it does not rely on perceptions based on actual mathematics ability. Instead, it seems that the self-confidence of siblings can be transmitted. The peer effects literature in economics has also highlighted the importance of non-cognitive peer effects over and above traditional cognitive peer effects (Golsteyn et al. (2021); Shure (2021)), which is in line with our finding.

We also find that the confidence of a male twin works the same way for self-assessed English and physical abilities as for SAMA. This is a striking result that highlights the importance of peer effects for self-confidence beyond one single domain and again without the support of actual ability. The confidence of a male twin is positively correlated with the confidence of brothers but not with the confidence of sisters even in English, where girls are better on average than boys. These sibling peer effects provide suggestive evidence on the formation of gender gaps in self-confidence, which can have implications across the lifecourse.

While again, our results are not causal, they might offer a potential explanation for the gender gap in labor market outcomes, especially in top jobs and high-level managerial positions. For women, exposure to highly confident men might be more off-putting than for men. As top job positions are traditionally filled by confident men, women might suffer a double penalty: not only are they less confident than men, as shown by Adamecz-Völgyi and Shure (2022), but their confidence is not supported in those environments (while men’s confidence might be). This phenomenon may serve as a barrier to both entry and progression for women in top jobs.

Our results are in line with the literature on the transmission of gendered stereotypes from adults to the next generation. Parental assessments of the mathematics performance of their children (conditional on objective skills) explain a further 23% of the gender gap in SAMA. This is larger than the share explained by objective ability. Furthermore, we find that most of the gender gap is driven by families where parents assess their children according to gender stereotypes, i.e., assess boys higher and girls lower in mathematics. For those children in families without stereotypical assessments, the gender gap in self-assessments is small. Again this highlights the importance of gender norms in how parents assess their children.

Unfortunately, teachers are not immune to this and also over-assess boys and under-assess girls; however, this explains a smaller portion of the gender gap in SAMA. We cannot exclude, however, two potential sources of endogeneity between parents’ and children’s assessments. First, parents might have some unobserved knowledge about the math abilities of their children, that is above and beyond their objective math levels and their teachers’ assessments, hence they play such an important role in terms of explaining the gender gap in SAMA. Second, parental assessments might mirror their children’s own assessment, hence they are so highly correlated. Identifying the causal effects of parental assessments/stereotypes on their children’s own assessments is an extremely challenging exercise that has not been solved yet.

Although we find that girls have a comparative advantage in English, this does not explain the gender gap in SAMA. Having higher English ability or higher self-assessed English ability does not reduce the gender gap in SAMA. Girls are not specializing in one domain at the expense of another.

There are potential explanations behind our findings that could not be explored in this paper. This includes disentangling in-utero testosterone exposure (Auyeung et al. (2009); Gielen et al. (2016)) from the environmental exposure of growing up with a brother. While we have looked at whether having a brother is different from having a twin brother, our setup does not allow for causal identification.

Our study also has some caveats. First, unobserved facets of mathematics ability, which might be known by kids/parents/teachers, but not measured by mathematics levels or cognitive skills could hinder our results. Despite our efforts to carry out various robustness checks around our measures of mathematics ability, they may be subject to some degree of measurement error. Reassuringly, when we replicated our main result using the age 12 data that also captured math test scores besides math levels, we found similar results. However, parental assessments are not available at age 12, so we could not test our results on the role of parental assessments.

Second, parents, teachers, and the twins were all asked to assess their mathematics ability in the same wave. As mentioned above, it may be the case that children’s self-assessments shape their parents’ or teachers’ assessments as much as the adults’ assessments shape the children’s. We unfortunately cannot account for the direction of this relationship since the parents and teachers were only asked about the twins’ mathematics ability in one wave. Third, confidence is not randomly assigned to individuals or their twins. Lab experiments are needed to test what happens to the gender gap in confidence when women/men are randomly exposed to more confident male/female peers.

In terms of policy, our results suggest that potential interventions to reduce the gender gap in SAMA should also target parents and teachers, not just children. It is not enough to inspire girls into STEM fields, systematic change around who adults frame as the “mathematics person” is also needed. Teacher training could include further emphasis on unconscious bias in marking and assessment. There are excellent examples of successful teacher training programs to foster general gender equity including REFLECT (Kollmayer et al. 2020), as well as STEM-specific gender equity programs, such as the UK’s Institute of Physics’ Opening Doors Program, which includes nine concrete action points for schools (of Physics 2015). This includes creating a gender champion in the senior leader team and reworking career guidance so that it is not based on gender stereotypes.

Parents should be aware of the narratives they develop within families to place children into “math” or “verbal” person categories as this early differentiation can have long-lasting consequences (Chaffee and Plante 2022). UNICEF has five suggestions on breaking parental gender stereotypes (UNICEF n.d.), which include not using gendered language and removing gendered toys from playtime. These could be promoted to parents to break the transmission of gender stereotypes. These types of changes are not easy and require every member of society to re-evaluate their role in facilitating the perpetuation and transmission of gender stereotypes to the next generation.

## Supplementary Information

Below is the link to the electronic supplementary material.

Supplementary file 1 (pdf 416 KB)

## Footnotes

1 All self-assessment questions are summarized in Table A1 in Appendix A.

2 Note that what we measure here is not girls being less confident on average than boys in general, but only in their SAMA. In self-assessed English abilities, for example, the gender gap is positive: girls assess themselves to be better than boys, even after controlling for objective abilities in English (Table O10 in the Online Appendix).

3 Only 1% of the sample has a younger brother, while among those who have brothers (32%), only 2.7% have a younger brother.

4 The number of observations in the triple interaction cell is *n* = 598, which is the number of female twins with a male co-twin. The third item of the tripe interaction, the SAMA of co-twin is continuous.

5 These results are the same for SAMA measured at age 12 after controlling for math test scores (Table B8 in Appendix B), using our IV strategies to correct for measurement error in math levels (Tables B9 and B10 in Appendix B) as well as after re-weighting the model with the three types of weights introduced above to account for selection to the analytical sample (Table O5 in the Online Appendix). When we restrict the sample to dizygotic twins in Table B11 in Appendix B, the estimated coefficient on the triple interaction is still negative, but smaller and insignificant. This is most likely the result of decreased sample size.

6 Theoretically, teachers could also show a gender bias when they determine the mathematics levels of kids. We tested on the age 12 sample whether there is a gender gap in mathematics levels. While there is a raw gender gap in mathematics levels, once mathematics test scores and cognitive abilities are controlled for, this gap becomes small and non-significant. We cannot test the same thing on the age nine sample as test scores are only available for age 12, but we believe that these results would be similar given the similar patterns that emerge using the age nine and age 12 data.

7 The measure of gender-stereotypical parental assessment is a binary variable that captures whether parents’ assessment of their children’s mathematics abilities is stereotypically gender-biased, i.e., they overestimate their son in mathematics and/or they underestimate their daughter in math.

8 As Table O15 in the Online Appendix shows, there is no gender gap in the probability of stereotypical evaluations, i.e., boys are just as likely to be overestimated in Math as girls to be underestimated. However, the returns to being stereotypically assessed are very different among girls than boys, hence our results.

9 Ideally, we would also want to look at the role of gender roles in the home using alternative measures. Unfortunately, the data do not include direct measures of gender roles. SAMA is neither correlated with parental education (Table O20 in the Online Appendix) nor with the characteristics of maternal employment (Table O21 in the Online Appendix). We have also tried to determine whether the relative educational or employment characteristics of mothers matter (i.e., if they have higher educational attainment or work in higher-status jobs than fathers), but they do not. We believe that the stereotypical assessment of their children’s mathematics skills is the best measure of parental gender stereotypes.
