# Supplementary material for: Peers, parents, and self-perceptions: the gender gap in mathematics self-assessment
Source: J Popul Econ. 2025 Feb 22;38(1):33. doi: 10.1007/s00148-025-01087-2 (PMC11846763; doi:10.1007/s00148-025-01087-2)
Supplement: Supplementary file 1 — (pdf 416 KB) [file 148_2025_1087_MOESM1_ESM.pdf]

Appendix: Peers, parents, and self-perceptions: The gender gap in mathematics self-assessment

Anna Adamecz[1], John Jerrim[2], Jean-Baptiste Pingault[3], and Nikki Shure[4]

[1]UCL Social Research Institute, KRTK KTI and IZA
[2]UCL Social Research Institute
[3]UCL Department of Clinical, Educational and Health Psychology and KCL Social, Genetic & Developmental Psychiatry Centre
[4]UCL Social Research Institute and IZA

Appendix

A Survey questions and descriptive statistics

Table A1: Assessment questions in TEDS, age 9

Mathematics	English	Physical Education
	Twins	
	How good do you think you are at:	
Solving number and money problems?	Reading?	Playing team games?
Doing maths in your head?	Writing?	Races and competitions?
Multiplying and dividing?	Spelling?	PE classes
Potential answers: very good; quite good; doing OK; not so good; not at all good		
	Parents	
	How good do you think your children are at:	
Solving number and money problems?	Reading?	Playing team games?
Doing mental arithmetic?	Writing?	Races and competitions?
Multiplying and dividing?	Spelling?	PE classes
Potential answers: very good; quite good; doing OK; not so good; not at all good		
	Teachers - subjective assessment	
	How good is this pupil at:	
Solving number and money problems?	Reading?	Playing team games?
Doing mental arithmetic?	Writing?	Races and competitions?
Multiplying and dividing?	Spelling?	PE classes
Potential answers: very poor; poor; satisfactory; very good, excellent		
	Teachers - objective assessment	
	National Curriculum Rating	
Using and applying Mathematics	Speaking and listening	
Number and algebra	Reading	
Shapes, space and Measures	Writing	
Potential answers: 1-2-3-4-5		

Source: TEDS (Rimfeld et al., 2019) questionnaires from https://www.teds.ac.uk/datadictionary/pdfs/booklets.htm.

Table A2: Descriptive statistics, age nine sample

	Mean	SD	Min	Max	N
Female	0.54	0.50	0.00	1.00	3,877
Cohort born between Jan 94-Aug 94	0.38	0.48	0.00	1.00	3,877
Cohort born between Sep 94-Aug 95	0.62	0.48	0.00	1.00	3,877
Self-assessed Math (SAMA), age 9	3.83	0.99	1.00	5.00	3,877
Math level, age 9	0.09	0.97	-2.94	2.99	3,877
Verbal abilities, age 9	0.06	0.96	-3.34	2.61	3,877
Non-verbal abilities, age 9	0.07	0.96	-3.72	1.39	3,877
Elder twin	0.50	0.50	0.00	1.00	3,877
Heavier twin at birth	0.47	0.50	0.00	1.00	3,877
Birthweight, gramms	2,536.82	546.79	595.88	6,320.00	3,877
Verbal abilities, age 7	0.10	0.98	-3.04	5.90	3,473
Non-verbal abilities, age 7	0.08	0.96	-3.64	2.53	3,487
Math level, age 7	0.05	0.93	-3.68	3.23	3,059
Self-assessed English (SAEA), age 9	4.11	0.70	1.00	5.00	3,877
English level, age 9	0.10	0.96	-3.08	3.07	3,877
Parental assessment of Math	3.94	0.93	1.00	5.00	3,877
Teachers' assessment of Math	3.37	0.83	1.00	5.00	3,877
Has a male twin (MT)	0.46	0.50	0.00	1.00	3,877
Has brother	0.32	0.47	0.00	1.00	3,877
Overestimated in Math	0.23	0.42	0.00	1.00	3,877
Underestimated in Math	0.23	0.42	0.00	1.00	3,877
Stereotipically assessed person	0.26	0.44	0.00	1.00	3,877
Stereotipically assessed person, 5 cat	0.32	0.47	0.00	1.00	3,877
No qual or low-grade CSE/GCSE	0.11	0.31	0.00	1.00	3,863
High-grade CSE/GCSE	0.31	0.46	0.00	1.00	3,863
A-level or below degree	0.28	0.45	0.00	1.00	3,863
Degree	0.31	0.46	0.00	1.00	3,863
Mother has A-levels or above	0.42	0.49	0.00	1.00	3,877
Mother has managerial job	0.11	0.31	0.00	1.00	3,877
Mother needs qualification	0.25	0.44	0.00	1.00	3,877
SAMA of CT, age 9	3.82	0.99	1.00	5.00	3,877
Math level of CT, age 9	0.08	0.97	-2.94	2.99	3,877
Self-assessed physical (SAPA), age 9	4.44	0.67	1.00	5.00	3,867
SAEA of CT, age 9	0.04	0.97	-4.28	1.29	3,876
SAPA of CT, age 9	0.02	0.98	-4.97	0.84	3,863

Source: TEDS (Rimfeld et al., 2019).

Table A3: The gender gap in our main measures, age nine sample

	boys	girls	gap	se	pvalue	obs
Verbal abilities, age 7	0.105	0.097	-0.009	0.037	0.815	3473
Non-verbal abilities, age 7	0.079	0.080	0.001	0.036	0.976	3487
Math level, age 7	0.078	0.023	-0.056	0.038	0.148	3059
Verbal abilities, age 9	0.083	0.037	-0.045	0.035	0.194	3877
Non-verbal abilities, age 9	0.058	0.079	0.021	0.035	0.539	3877
Math level, age 9	0.157	0.029	-0.129	0.035	0.000	3877
Self-assessed Math (SAMA), age 9, std	0.204	-0.173	-0.377	0.034	0.000	3877
English level, age 9	-0.035	0.221	0.257	0.035	0.000	3877
Self-assessed English (SAEA), age 9, std	-0.121	0.103	0.224	0.035	0.000	3877
Self-assessed physical (SAPA), age 9	0.069	-0.014	-0.083	0.034	0.014	3867
Parental assessment of Math, std	0.157	-0.134	-0.291	0.037	0.000	3877
Teachers' assessment of Math, std	0.121	-0.103	-0.224	0.036	0.000	3877
Verbal abilities, age 12	0.372	0.108	-0.263	0.042	0.000	2469
Non-verbal abilities, age 12	0.337	0.286	-0.052	0.042	0.219	2398
SAMA, age 12, std	0.324	-0.081	-0.405	0.089	0.000	507
Math level, age 12	0.506	0.456	-0.050	0.051	0.331	1497
Math test scores, age 12, std	0.485	0.213	-0.272	0.087	0.002	507
Overestimated in Math	0.266	0.209	-0.057	0.015	0.000	3877
Underestimated in Math	0.195	0.264	0.069	0.015	0.000	3877
Stereotipically assessed person	0.266	0.264	-0.002	0.016	0.915	3877
Stereotipically assessed person, 5 cat	0.230	0.394	0.163	0.017	0.000	3877

Source: TEDS (Rimfeld et al., 2019).

Table A4: Correlation matrix of measures, age nine

	(1)	(2)	(3)	(4)	(5)	(6)	(7)	(8)	(9)	(10)
(1) SAMA	1	0.22	0.37	0.17	0.29	0.20	0.19	0.20	0.54	0.42
(2) SAMA of co-twin	0.22	1	0.17	0.37	0.15	0.12	0.11	0.10	0.20	0.19
(3) Math level	0.37	0.17	1	0.53	0.23	0.72	0.33	0.38	0.58	0.78
(4) Math level of co-twin	0.17	0.37	0.53	1	0.13	0.47	0.23	0.26	0.31	0.44
(5) Self-assessed English	0.29	0.15	0.23	0.13	1	0.35	0.19	0.11	0.20	0.22
(6) English levels	0.20	0.12	0.72	0.47	0.35	1	0.34	0.33	0.41	0.59
(7) Verbal abilities	0.19	0.11	0.33	0.23	0.19	0.34	1	0.40	0.29	0.30
(8) Non-verbal abilities	0.20	0.10	0.38	0.26	0.11	0.33	0.40	1	0.33	0.36
(9) Parental assessment	0.54	0.20	0.58	0.31	0.20	0.41	0.29	0.33	1	0.61
(10) Teachers assessment	0.42	0.19	0.78	0.44	0.22	0.59	0.30	0.36	0.61	1

Source: TEDS (Rimfeld et al., 2019). Number of observations: 3,877.

Figure A1: The distribution of mathematics self-assessment, age 12

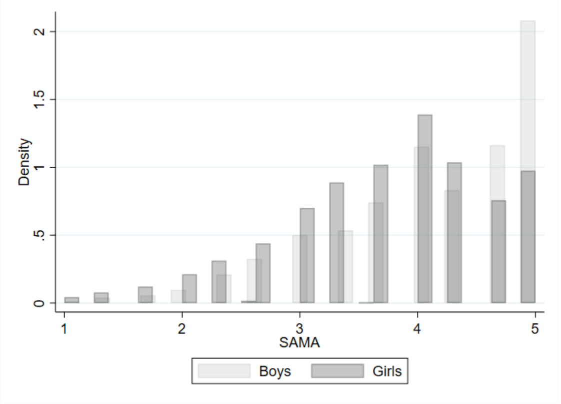

Source: TEDS (Rimfeld et al., 2019). Number of observations: 3,196.

Figure A2: The distribution of mathematics levels, age 12

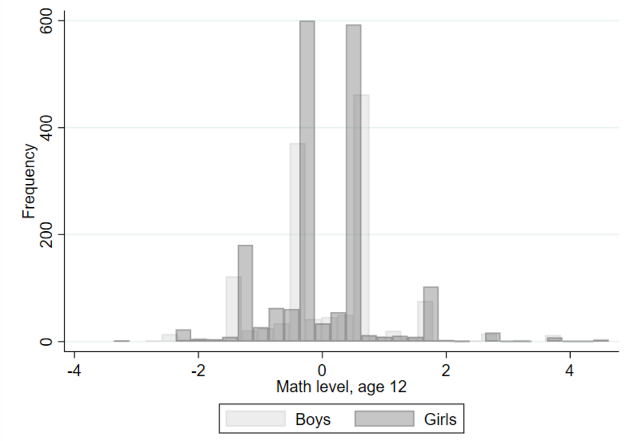

Source: TEDS (Rimfeld et al., 2019). Number of observations: 3,196.

Figure A3: The distribution of mathematics test scores, age 12

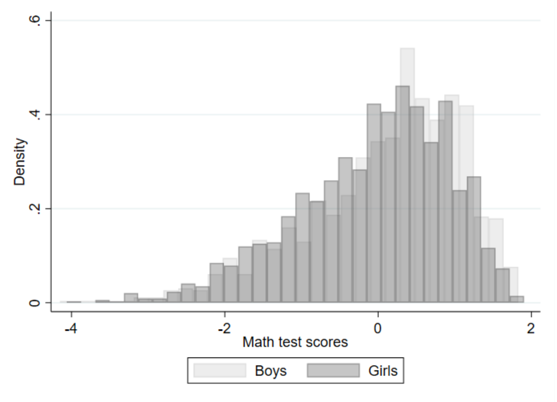

Source: TEDS (Rimfeld et al., 2019). Number of observations: 3,196.

B Robustness checks

Table B1: The gender gap in SAMA at age nine - controlling for math levels and abilities from age seven

VARIABLES	(1)<br>Model 1	(2)<br>Model 2	(3)<br>Model 3	(4)<br>Model 4	(5)<br>Model 5	(6)<br>Model 6
Female	-0.328***	-0.310***	-0.307***	-0.449***	-0.506***	-0.491***
	(0.032)	(0.036)	(0.036)	(0.052)	(0.060)	(0.059)
Math level, age 9		0.333***	0.284***		0.363***	0.309***
		(0.021)	(0.025)		(0.038)	(0.041)
Math level, age 7			0.132***			0.197***
			(0.025)			(0.044)
Verbal abilities, age 9	0.063***	0.062***	0.062***	0.095***	0.114***	0.096**
	(0.018)	(0.021)	(0.022)	(0.036)	(0.043)	(0.043)
Non-verbal abilities, age 9	0.070***	0.070***	0.055**	0.148***	0.130***	0.107***
	(0.020)	(0.023)	(0.023)	(0.033)	(0.040)	(0.039)
Verbal abilities, age 7			-0.067***			-0.084**
			(0.021)			(0.036)
Non-verbal abilities, age 7			0.024			0.058*
			(0.020)			(0.031)
Math level = 2	0.443***			0.446***		
	(0.045)			(0.069)		
Math level = 3	0.824***			0.888***		
	(0.050)			(0.085)		
Constant	-0.499***	-0.112	-0.091	-0.416*	0.157	0.112
	(0.094)	(0.102)	(0.102)	(0.243)	(0.274)	(0.272)
Observations	3,877	2,942	2,942	3,877	2,942	2,942
R-squared	0.164	0.175	0.186	0.156	0.177	0.195
Twin FE	No	No	No	Yes	Yes	Yes
Cohort FE	Yes	Yes	Yes	No	No	No

Source: TEDS (Rimfeld et al., 2019). Robust standard errors clustered within twin pairs in parentheses. *** p<0.01, ** p<0.05, * p<0.1 Further control variables: elder twin, heavier twin, and birth weight.

Table B2: The gender gap in SAMA at age nine - using age seven math levels as an IV for age nine math levels

VARIABLES	(1)<br>Model 1	(2)<br>Model 2	(3)<br>Model 3	(4)<br>Model 3 OS subsample	(5)<br>Model 4
Female	-0.352***	-0.297***	-0.297***	-0.454***	-0.457***
	(0.039)	(0.037)	(0.037)	(0.059)	(0.063)
Math level, age 9		0.549***	0.475***	0.499***	0.717***
		(0.035)	(0.043)	(0.073)	(0.110)
Verbal abilities, age 9			0.032	-0.060	0.040
			(0.023)	(0.037)	(0.049)
Non-verbal abilities, age 9			0.027	0.043	0.040
			(0.026)	(0.041)	(0.048)
Constant	0.174***	0.055	-0.089	0.197	0.108
	(0.040)	(0.038)	(0.103)	(0.166)	(0.280)
Observations	2,942	2,942	2,942	901	2,942
R-squared	0.031	0.139	0.160	0.189	
Twin FE	No	No	No	No	Yes
Cohort FE	Yes	Yes	Yes	Yes	No

Source: TEDS (Rimfeld et al., 2019). Robust standard errors clustered within twin pairs in parentheses. *** p<0.01, ** p<0.05, * p<0.1 Further control variables: elder twin, heavier twin, and birth weight. 2SLS estimates using age seven math levels as instrumental variables for age nine math levels.

Table B3: The gender gap in SAMA at age nine - using the ORIV approach of Gillen et al. (2019)

VARIABLES	(1)<br>Model 1	(2)<br>Model 2	(3)<br>Model 3	(4)<br>Model 3 OS subsample	(5)<br>Model 4
Female	-0.347***	-0.298***	-0.291***	-0.413***	-0.604***
	(0.039)	(0.036)	(0.036)	(0.058)	(0.075)
Math levels		0.574***	0.568***	0.583***	-0.601***
		(0.030)	(0.039)	(0.061)	(0.097)
Verbal abilities, age 9			0.018	-0.088**	0.313***
			(0.022)	(0.036)	(0.056)
Non-verbal abilities, age 9			-0.012	0.000	0.354***
			(0.025)	(0.040)	(0.053)
Constant	0.165***	0.129***	0.006	0.193	0.208
	(0.039)	(0.037)	(0.102)	(0.167)	(0.338)
Observations	6,118	6,118	6,118	1,874	6,118
R-squared	0.031	0.081	0.085	0.117	
Twin FE	No	No	No	No	Yes
Cohort FE	Yes	Yes	Yes	Yes	No

Source: TEDS (Rimfeld et al., 2019). Robust standard errors clustered within twin pairs in parentheses. *** p<0.01, ** p<0.05, * p<0.1 Further control variables: elder twin, heavier twin, and birth weight. 2SLS estimates using the ORIV approach of Gillen et al. (2019).

Table B4: The gender gap in SAMA at age nine - excluding monozygotic twins from the sample

VARIABLES	(1)<br>Model 1	(2)<br>Model 2	(3)<br>Model 3	(4)<br>Model 3 OS subsample	(5)<br>Model 4
Female	-0.445***	-0.384***	-0.378***	-0.449***	-0.447***
	(0.040)	(0.037)	(0.037)	(0.051)	(0.052)
Math level, age 9		0.374***	0.336***	0.319***	0.395***
		(0.019)	(0.021)	(0.030)	(0.036)
Verbal abilities, age 9			0.028	-0.030	0.047
			(0.022)	(0.030)	(0.044)
Non-verbal abilities, age 9			0.071***	0.110***	0.140***
			(0.024)	(0.034)	(0.041)
Constant	0.249***	0.170***	0.022	0.251*	0.202
	(0.040)	(0.037)	(0.109)	(0.151)	(0.317)
Observations	2,436	2,436	2,436	1,186	2,436
R-squared	0.050	0.183	0.190	0.195	0.211
Twin FE	No	No	No	No	Yes
Cohort FE	Yes	Yes	Yes	Yes	No

Source: TEDS (Rimfeld et al., 2019). Sample of dizygotic twins only. Robust standard errors clustered within twin pairs in parentheses. *** p<0.01, ** p<0.05, * p<0.1 Further control variables: elder twin, heavier twin, and birth weight.

Table B5: The gender gap in SAMA at age 12, OLS models

VARIABLES	(1) Model 1	(2) Model 2	(3) Model 3	(4) Model 4	(5) Model 5
Female	-0.394***	-0.337***	-0.299***	-0.340***	-0.285***
	(0.038)	(0.034)	(0.033)	(0.075)	(0.082)
Math level, age 12		0.459***	0.296***	0.228***	0.184***
		(0.023)	(0.023)	(0.048)	(0.054)
Math test scores, age 12			0.022***	0.014***	0.012***
			(0.002)	(0.004)	(0.004)
Verbal abilities, age 12			-0.008	-0.026	-0.068
			(0.020)	(0.047)	(0.057)
Non-verbal abilities, age 12			-0.015	0.044	0.047
			(0.020)	(0.049)	(0.050)
Math level, age 9				0.216***	0.191***
				(0.045)	(0.055)
Verbal abilities, age 9				0.037	0.018
				(0.041)	(0.045)
Non-verbal abilities, age 9				0.059	0.100*
				(0.049)	(0.056)
Math level, age 7					0.125**
					(0.059)
Verbal abilities, age 7					0.046
					(0.053)
Non-verbal abilities, age 7					-0.020
					(0.046)
Elder twin			0.029	0.006	0.030
			(0.027)	(0.062)	(0.067)
Heavier twin at birth			0.024	0.128*	0.131*
			(0.030)	(0.067)	(0.073)
Birth weight, grams			0.000	-0.000	-0.000
			(0.000)	(0.000)	(0.000)
Constant	0.272***	-0.004	-1.649***	-0.871***	-0.792**
	(0.058)	(0.054)	(0.142)	(0.322)	(0.335)
Observations	3,196	3,196	3,196	570	460
R-squared	0.038	0.205	0.263	0.343	0.361
Twin FE	No	No	No	No	No
Cohort FE	Yes	Yes	Yes	Yes	Yes

Source: TEDS (Rimfeld et al., 2019). Robust standard errors clustered within twin pairs in parentheses. *** p<0.01, ** p<0.05, * p<0.1

Table B6: The gender gap in SAMA at age 12 - FE models

VARIABLES	(1) Model 1	(2) Model 2	(3) Model 3	(4) Model 4	(5) Model 5
Female	-0.482***	-0.448***	-0.413***	-0.536***	-0.464***
	(0.065)	(0.060)	(0.060)	(0.134)	(0.138)
Math level, age 12		0.547***	0.420***	0.284***	0.148*
		(0.043)	(0.042)	(0.076)	(0.084)
Math test scores, age 12			0.019***	0.018**	0.012
			(0.003)	(0.007)	(0.008)
Verbal abilities, age 12			0.069**	0.016	-0.065
			(0.034)	(0.094)	(0.100)
Non-verbal abilities, age 12			-0.006	0.093	0.087
			(0.031)	(0.067)	(0.069)
Math level, age 9				0.247***	0.226**
				(0.079)	(0.090)
Verbal abilities, age 9				0.016	-0.009
				(0.083)	(0.098)
Non-verbal abilities, age 9				-0.070	-0.048
				(0.089)	(0.108)
Math level, age 7					0.342***
					(0.113)
Verbal abilities, age 7					0.192**
					(0.078)
Non-verbal abilities, age 7					-0.057
					(0.070)
Elder twin			0.030	-0.031	0.010
			(0.028)	(0.065)	(0.071)
Heavier twin at birth			0.045	0.179*	0.083
			(0.047)	(0.105)	(0.115)
Birth weight, grams			-0.000	-0.000	-0.000
			(0.000)	(0.000)	(0.000)
Constant	0.277***	0.225***	-1.012***	-0.307	-0.527
	(0.037)	(0.035)	(0.338)	(0.702)	(0.712)
Observations	3,196	3,196	3,196	570	460
R-squared	0.043	0.178	0.224	0.301	0.321
Twin FE	Yes	Yes	Yes	Yes	Yes
Cohort FE	No	No	No	No	No

Source: TEDS (Rimfeld et al., 2019). Robust standard errors clustered within twin pairs in parentheses. *** p<0.01, ** p<0.05, * p<0.1

Table B7: SAMA as a categorical variable, age nine (Multinomial logit model)

VARIABLES	(1)<br>SAMA=1	(2)<br>SAMA=2	(3)<br>SAMA=3	(4)<br>SAMA=4	(5)<br>SAMA=5
Female	0.251	0.107		-0.398***	-1.021***
	(0.180)	(0.121)		(0.085)	(0.109)
Math level, age 9	-0.723***	-0.283***		0.353***	0.757***
	(0.102)	(0.068)		(0.049)	(0.066)
Verbal abilities, age 9	-0.096	-0.116*		0.058	0.070
	(0.092)	(0.066)		(0.047)	(0.063)
Non-verbal abilities, age 9	-0.162	-0.079		0.038	0.096
	(0.099)	(0.065)		(0.048)	(0.066)
Constant	-2.274***	-1.161***		0.109	-0.738**
	(0.510)	(0.313)		(0.227)	(0.287)
Observations	3,877	3,877	3,877	3,877	3,877

Source: TEDS (Rimfeld et al., 2019). Robust standard errors clustered within twin pairs in parentheses. *** p<0.01, ** p<0.05, * p<0.1 The estimated multinomial logit model is the following: $f(k,i) = beta_k * x_i$, where $beta_k$ is a set of regression coefficients associated with (integer) SAMA values k, $k = 1, 2, .., 5$, and x_i is the same set of explanatory variables associated with observation i as before. $SAMA = 3$ is the baseline category. Further control variables: mathematics level at age nine, verbal and non-verbal cognitive skills at age nine, elder twin, heavier twin, and birth weight.

Table B8: The role of co-twin (CT) SAMA, age 12 sample

	(1)	(2)	(3)	(4)	(5)	(6)
					Model 4	Model 4
VARIABLES	Model 1	Model 2	Model 3	Model 4	boys	girls
Female	-0.279***	-0.280***	-0.345***	-0.316***		
	(0.033)	(0.033)	(0.039)	(0.036)		
Has a male twin (MT)			-0.156***	-0.140***	-0.116**	-0.164***
			(0.040)	(0.037)	(0.051)	(0.052)
SAMA of CT, age 12, std	0.132***	0.117***	0.139***	0.029	0.023	0.181***
	(0.024)	(0.032)	(0.029)	(0.044)	(0.043)	(0.036)
MT*SAMA of CT			0.015	0.163***	0.169***	-0.098
			(0.038)	(0.059)	(0.060)	(0.060)
Female*SAMA of CT		0.027		0.150***		
		(0.037)		(0.054)		
Female*MT*SAMA of CT				-0.266***		
				(0.103)		
Constant	-1.499***	-1.500***	-1.403***	-1.414***	-1.449***	-1.691***
	(0.141)	(0.141)	(0.143)	(0.141)	(0.213)	(0.183)
Observations	3,012	3,012	3,012	3,012	1,275	1,737
R-squared	0.279	0.279	0.283	0.287	0.279	0.252
Twin FE	No	No	No	No	No	No
Cohort FE	Yes	Yes	Yes	Yes	Yes	Yes

Notes: Source: TEDS (Rimfeld et al., 2019). Robust standard errors clustered within twin pairs in parentheses. *** p<0.01, ** p<0.05, * p<0.1. Further control variables: mathematics level and mathematics test scores at age 12, verbal and non-verbal cognitive skills at age 12, elder twin, heavier twin, and birth weight.

Table B9: The role of co-twin (CT) SAMA - using age seven math levels as an IV for age nine math levels

	(1)	(2)	(3)	(4)	(5)	(6)
VARIABLES	Model 1	Model 2	Model 3	Model 4	Model 4 boys	Model 4 girls
Female	-0.305***	-0.305***	-0.373***	-0.331***		
	(0.035)	(0.036)	(0.039)	(0.038)		
Math level, age 9	0.431***	0.431***	0.425***	0.419***	0.445***	0.383***
	(0.043)	(0.043)	(0.042)	(0.042)	(0.060)	(0.057)
Has a male twin (MT)			-0.177***	-0.149***	-0.177***	-0.120**
			(0.041)	(0.037)	(0.053)	(0.053)
SAMA of CT, age 9, std	0.129***	0.100***	0.167***	0.015	0.019	0.231***
	(0.026)	(0.034)	(0.033)	(0.043)	(0.043)	(0.042)
MT*SAMA of CT			-0.050	0.147**	0.140**	-0.226***
			(0.044)	(0.062)	(0.062)	(0.065)
Female*SAMA of CT		0.055		0.212***		
		(0.042)		(0.058)		
Female*MT*SAMA of CT				-0.362***		
				(0.107)		
Constant	-0.076	-0.073	0.037	0.011	0.029	-0.314**
	(0.096)	(0.097)	(0.099)	(0.098)	(0.138)	(0.125)
Observations	2,824	2,824	2,824	2,824	1,296	1,528
R-squared	0.182	0.183	0.190	0.198	0.184	0.162
Twin FE	No	No	No	No	No	No
Cohort FE	Yes	Yes	Yes	Yes	Yes	Yes

Notes: Source: TEDS (Rimfeld et al., 2019). Robust standard errors clustered within twin pairs in parentheses. *** p<0.01, ** p<0.05, * p<0.1. Further control variables: mathematics level and mathematics test scores at age 12, verbal and non-verbal cognitive skills at age 12, elder twin, heavier twin, and birth weight. 2SLS estimates using age seven math levels as instrumental variables for age nine math levels.

Table B10: The role of co-twin (CT) SAMA - using the ORIV approach of Gillen et al. (2019)

VARIABLES	(1)<br>Model 1	(2)<br>Model 2	(3)<br>Model 3	(4)<br>Model 4	(5)<br>Model 4<br>boys	(6)<br>Model 4<br>girls
Female	-0.295***	-0.295***	-0.351***	-0.312***		
	(0.035)	(0.035)	(0.039)	(0.037)		
Math levels	0.529***	0.529***	0.524***	0.518***	0.502***	0.536***
	(0.040)	(0.040)	(0.040)	(0.040)	(0.052)	(0.060)
Has a male twin (MT)			-0.147***	-0.122***	-0.130**	-0.115**
			(0.040)	(0.037)	(0.052)	(0.053)
SAMA of CT, age 9, std	0.114***	0.086***	0.152***	0.007	0.010	0.206***
	(0.026)	(0.033)	(0.032)	(0.043)	(0.043)	(0.042)
MT*SAMA of CT			-0.056	0.134**	0.134**	-0.217***
			(0.043)	(0.061)	(0.061)	(0.064)
Female*SAMA of CT		0.053		0.201***		
		(0.041)		(0.058)		
Female*MT*SAMA of CT				-0.349***		
				(0.104)		
Constant	0.008	0.010	0.100	0.073	0.059	-0.220*
	(0.097)	(0.097)	(0.099)	(0.097)	(0.134)	(0.127)
Observations	5,876	5,876	5,876	5,876	2,696	3,180
R-squared	0.113	0.114	0.121	0.130	0.129	0.079
Twin FE	No	No	No	No	No	No
Cohort FE	Yes	Yes	Yes	Yes	Yes	Yes

Notes: Source: TEDS (Rimfeld et al., 2019). Robust standard errors clustered within twin pairs in parentheses. *** p<0.01, ** p<0.05, * p<0.1. Further control variables: mathematics level and mathematics test scores at age 12, verbal and non-verbal cognitive skills at age 12, elder twin, heavier twin, and birth weight. 2SLS estimates using the ORIV approach of Gillen et al. (2019).

Table B11: The role of co-twin (CT) SAMA - excluding monozygotic twins from the sample

VARIABLES	(1)<br>Model 1	(2)<br>Model 2	(3)<br>Model 3	(4)<br>Model 4	(5)<br>Model 4<br>boys	(6)<br>Model 4<br>girls
Female	-0.390***	-0.392***	-0.393***	-0.382***		
	(0.038)	(0.038)	(0.038)	(0.039)		
Has a male twin (MT)			-0.088**	-0.085**	-0.052	-0.117**
			(0.039)	(0.038)	(0.054)	(0.055)
SAMA of CT, age 9, std	0.053**	0.032	0.082**	0.035	0.030	0.128**
	(0.027)	(0.032)	(0.032)	(0.036)	(0.036)	(0.051)
MT*SAMA of CT			-0.042	0.016	0.011	-0.093
			(0.041)	(0.067)	(0.068)	(0.067)
Female*SAMA of CT		0.041		0.088		
		(0.039)		(0.061)		
Female*MT*SAMA of CT				-0.110		
				(0.107)		
Constant	0.029	0.032	0.068	0.066	0.029	-0.286*
	(0.109)	(0.109)	(0.110)	(0.110)	(0.148)	(0.150)
Observations	2,328	2,328	2,328	2,328	1,119	1,209
R-squared	0.195	0.195	0.197	0.198	0.178	0.141
Twin FE	No	No	No	No	No	No
Cohort FE	Yes	Yes	Yes	Yes	Yes	Yes

Notes: Source: TEDS (Rimfeld et al., 2019). Sample of dizygotic twins only. Robust standard errors clustered within twin pairs in parentheses. *** p<0.01, ** p<0.05, * p<0.1 Further control variables: mathematics level at age nine, verbal and non-verbal cognitive skills at age nine, elder twin, heavier twin, and birth weight. CT refers to co-twins.

Table B12: The role of stereotypically gender-biased parental assessments in the gender gap in SAMA - alternative model

	(1)	(2)	(3)	(4)	(5)	(6)
VARIABLES	Model 1	Model 2	Model 3	Model 4	Model 5	Model 6
Female	-0.324***	-0.303***	0.033	-0.447***	-0.442***	-0.052
	(0.032)	(0.032)	(0.037)	(0.051)	(0.052)	(0.061)
Stereotipically assessed person, 5 cat		-0.127***	0.577***		-0.048	0.586***
		(0.034)	(0.045)		(0.051)	(0.080)
Female*stereotipically assessed, 5 cat			-1.144***			-1.137***
			(0.063)			(0.111)
Constant	-0.064	-0.035	-0.114	0.033	0.040	-0.069
	(0.089)	(0.089)	(0.085)	(0.236)	(0.237)	(0.227)
Observations	3,877	3,877	3,877	3,877	3,877	3,877
R-squared	0.174	0.177	0.240	0.164	0.164	0.216
Twin FE	No	No	No	Yes	Yes	Yes
Cohort FE	Yes	Yes	Yes	No	No	No

Notes: Source: TEDS (Rimfeld et al., 2019). Robust standard errors clustered within twin pairs in parentheses. *** p<0.01, ** p<0.05, * p<0.1 Further control variables: mathematics levels at age nine, verbal and non-verbal cognitive skills at age nine, elder twin, heavier twin, and birth weight. The alternative measure of parental stereotypical assessment was created the following way. First, a multinomial logit model of the form $f(k,i) = beta_k * x_i$ is estimated, where $beta_k$ is a set of regression coefficients associated with k categories of parental assessments, $k = 1, 2, 3, 4, 5$, and x_i is the set of three explanatory variables: mathematics levels and verbal and non-verbal cognitive skills at age nine. Then, predicted categories of parental assessment are fitted by the model and they are compared to the observed parental assessment of individuals. An individual is over(under)estimated if their observed parental assessment category is higher(lower) than their predicted category.

Table B13: The role of stereotypically gender-biased parental assessments in the gender gap in SAMA - using age seven math levels as an IV for age nine math levels

VARIABLES	(1) Model 1	(2) Model 2	(3) Model 3	(4) Model 4	(5) Model 5	(6) Model 6
Female	-0.297***	-0.297***	-0.001	-0.457***	-0.458***	-0.112
	(0.037)	(0.037)	(0.043)	(0.063)	(0.062)	(0.074)
Math level, age 9	0.475***	0.475***	0.461***	0.717***	0.719***	0.675***
	(0.043)	(0.043)	(0.041)	(0.110)	(0.110)	(0.103)
Stereotipically assessed person		-0.005	0.619***		-0.034	0.561***
		(0.041)	(0.051)		(0.065)	(0.092)
Female*stereotipically assessed			-1.157***			-1.143***
			(0.082)			(0.144)
Constant	-0.089	-0.088	-0.196**	0.108	0.109	-0.043
	(0.103)	(0.104)	(0.099)	(0.280)	(0.280)	(0.267)
Observations	2,942	2,942	2,942	2,942	2,942	2,942
R-squared	0.160	0.160	0.225			
Twin FE	No	No	No	Yes	Yes	Yes
Cohort FE	Yes	Yes	Yes	No	No	No

Notes: Source: TEDS (Rimfeld et al., 2019). Robust standard errors clustered within twin pairs in parentheses. *** p<0.01, ** p<0.05, * p<0.1 Further control variables: mathematics levels at age nine, verbal and non-verbal cognitive skills at age nine, elder twin, heavier twin, and birth weight. 2SLS estimates using age seven math levels as instrumental variables for age nine math levels. The measure of parental stereotypical assessment was created the following way. First, a multinomial logit model of the form $f(k,i) = beta_k * x_i$ is estimated, where $beta_k$ is a set of regression coefficients associated with the k terciles of parental assessments, $k = 1, 2, 3$, and x_i is the set of three explanatory variables: mathematics levels and verbal and non-verbal cognitive skills at age nine. Then, predicted categories of parental assessment are fitted by the model and they are compared to the observed parental assessment of individuals. An individual is over(under)estimated if their observed parental assessment category is higher(lower) than their predicted category.

Table B14: The role of stereotypically gender-biased parental assessments in the gender gap in SAMA - using the ORIV approach of Gillen et al. (2019)

VARIABLES	(1) Model 1	(2) Model 2	(3) Model 3	(4) Model 4	(5) Model 5	(6) Model 6
Female	-0.291***	-0.291***	-0.036	-0.604***	-0.600***	-0.346***
	(0.036)	(0.036)	(0.043)	(0.075)	(0.075)	(0.090)
Math levels	0.568***	0.569***	0.598***	-0.601***	-0.594***	-0.601***
	(0.039)	(0.039)	(0.039)	(0.097)	(0.096)	(0.097)
Stereotipically assessed person		-0.010	0.515***		0.131*	0.569***
		(0.038)	(0.050)		(0.073)	(0.113)
Female*stereotipically assessed			-0.985***			-0.845***
			(0.075)			(0.165)
Constant	0.006	0.009	-0.069	0.208	0.198	0.083
	(0.102)	(0.103)	(0.100)	(0.338)	(0.338)	(0.333)
Observations	6,118	6,118	6,118	6,118	6,118	6,118
R-squared	0.085	0.085	0.114			
Twin FE	No	No	No	Yes	Yes	Yes
Cohort FE	Yes	Yes	Yes	No	No	No

Notes: Source: TEDS (Rimfeld et al., 2019). Robust standard errors clustered within twin pairs in parentheses. *** p<0.01, ** p<0.05, * p<0.1 Further control variables: mathematics levels at age nine, verbal and non-verbal cognitive skills at age nine, elder twin, heavier twin, and birth weight. 2SLS estimates using age seven math levels as instrumental variables for age nine math levels. 2SLS estimates using the ORIV approach of Gillen et al. (2019). The measure of parental stereotypical assessment was created the following way. First, a multinomial logit model of the form $f(k,i) = beta_k * x_i$ is estimated, where $beta_k$ is a set of regression coefficients associated with the k terciles of parental assessments, $k = 1, 2, 3$, and x_i is the set of three explanatory variables: mathematics levels and verbal and non-verbal cognitive skills at age nine. Then, predicted categories of parental assessment are fitted by the model and they are compared to the observed parental assessment of individuals. An individual is over(under)estimated if their observed parental assessment category is higher(lower) than their predicted category.

Table B15: The role of stereotypically gender-biased parental assessments in the gender gap in SAMA - excluding monozygotic twins from the sample

	(1)	(2)	(3)	(4)	(5)	(6)
VARIABLES	Model 1	Model 2	Model 3	Model 4	Model 5	Model 6
Female	-0.378***	-0.378***	-0.047	-0.447***	-0.446***	-0.060
	(0.037)	(0.038)	(0.045)	(0.052)	(0.052)	(0.065)
Stereotypically assessed person		-0.000	0.572***		0.035	0.638***
		(0.040)	(0.051)		(0.059)	(0.084)
Female*stereotypically assessed			-1.165***			-1.236***
			(0.082)			(0.134)
Constant	0.022	0.022	-0.056	0.202	0.191	-0.000
	(0.109)	(0.110)	(0.106)	(0.317)	(0.318)	(0.301)
Observations	2,436	2,436	2,436	2,436	2,436	2,436
R-squared	0.190	0.190	0.253	0.211	0.211	0.271
Twin FE	No	No	No	Yes	Yes	Yes
Cohort FE	Yes	Yes	Yes	No	No	No

Notes: Source: TEDS (Rimfeld et al., 2019). Sample of dizygotic twins only.Robust standard errors clustered within twin pairs in parentheses. *** p<0.01, ** p<0.05, * p<0.1 Further control variables: mathematics levels at age nine, verbal and non-verbal cognitive skills at age nine, elder twin, heavier twin, and birth weight. The measure of parental stereotypical assessment was created the following way. First, a multinomial logit model of the form $f(k,i) = beta_k * x_i$ is estimated, where $beta_k$ is a set of regression coefficients associated with the k terciles of parental assessments, $k = 1, 2, 3$, and x_i is the set of three explanatory variables: mathematics levels and verbal and non-verbal cognitive skills at age nine. Then, predicted categories of parental assessment are fitted by the model and they are compared to the observed parental assessment of individuals. An individual is over(under)estimated if their observed parental assessment category is higher(lower) than their predicted category.

C Deviations from pre-registration protocol

As mentioned in the acknowledgments to the paper, this study was pre-registered in the OSF Registries (https://osf.io/chv5g). This is a pre-requisite of obtaining TEDS data and must be completed before data access is granted.

The analysis in this paper deviates from the pre-registration in three key ways. The first deviation is that we restricted the focus of the study from ambition, risk-taking, and overconfidence to overconfidence. This was due to the volume of results and the desire to keep the paper simple.

The second is that we decided not to construct the composite measure of overconfidence and instead focus just on mathematics self-assessment controlling for actual mathematics ability. In the psychological literature, there are two main ways of capturing overconfidence. One is to construct an overconfidence measure (either a residual score or a difference measure) using measures of self-assessment and actual ability. The other is to compare self-assessments conditional on actual ability. We had initially wanted to construct a similar overconfidence measure to Adamecz-Völgyi and Shure (2022), but TEDS did not have the range of measures to do this. The overconfidence measure we could have constructed would have been based only on English and mathematics self-assessments and on English and mathematics national curriculum levels (actual performance). Given that the gender and ability gaps work in opposite directions with these two measures, we would have ended up with an overconfidence measure that had zero gender differences. We decided instead to follow the second approach and look at conditional mathematics self-assessments since this was the category with the largest gender gap in favor of boys and the domain most important for future labor market success.

The third deviation is that we did not undertake the Kitagawa-Blinder-Oaxaca decomposition, but instead focused on linear regressions to assess the gender gap. Both of these methods were outlined in the protocol, but in the interest of brevity, we focus on the linear regression results.

Online Appendix to “Peers, parents, and self-perceptions: The gender gap in mathematics self-assessment”

O1 Attrition and non-response

As detailed in the paper, our main analytical sample contains 3,877 observations out of the total initial sample of Cohort (1) and (2) of TEDS (15,216 observations). In this section of the Online Appendix, we provide robustness checks to show that selection to this subsample of TEDS is not likely to bias our results.

Table O1 compares those in our analytical sample to those who participated in the first wave, but either dropped out by age nine or they did not provide all data we needed. Those in our analytical sample come from slightly better social backgrounds: their parents are more likely to have qualifications, work in better jobs, and their fathers were more likely to live with the family right after when they were born.

Table O2 looks at selection to the analytic sample using a linear probability and a probit model. SES is positively correlated with the probability of being in the sample, while missing data (i.e., non-response to some questions already in the first wave) is negatively correlated with it. Interestingly, those with younger siblings are also less likely to be in the sample.

Table O1: The differences between those in the analytical sample and those who dropped out

	Mean, dropouts	Mean, analytical sample	Diff	p-value
No father in family	0.1	0.06	0.05	0
Qual needed for job, mother	0.17	0.25	-0.09	0
Qual needed for job, father	0.39	0.47	-0.09	0
Family SES score	-0.13	0.17	-0.3	0
Family SES missing	0.1	0.04	0.06	0
Age of mother	30.28	31.38	-1.1	0
Age of mother missing	0.02	0.01	0.01	0
Cohort: 2	1.59	1.62	-0.03	0
Has younger siblings	0.03	0.02	0.02	0
Has older siblings	0.54	0.5	0.04	0
Father no qual	0.14	0.09	0.05	0
Mother no qual	0.12	0.06	0.07	0
Mother's qual missing	0	0	0	0
Emp of mother: manager	0.06	0.09	-0.03	0
Emp of mother: employee	0.28	0.33	-0.05	0
Emp of mother: SE with emps	0.02	0.01	0	0.237
Emp of mother: SE	0.03	0.04	-0.01	0.003
Emp of father: manager	0.22	0.29	-0.07	0
Emp of father: employee	0.36	0.38	-0.02	0.003
Emp of father: SE with emps	0.07	0.08	0	0.443
Emp of father: SE	0.1	0.1	0	0.265
Emp of father: Foreman	0	0	0	0.072
Ethnicity: White	0.9	0.95	-0.05	0
Ethnicity: Missing	0	0	0	0.026

Source: TEDS (Rimfeld et al., 2019). Robust standard errors clustered within twin pairs in parentheses. *** p«0.01, ** p«0.05, * p«0.1 Sample of those in the first wave. No. of observations: 3,877 in the analytical sample, 11,339 dropped out or did not provide all data at age nine. Note that there are also differences between the two groups in their ACORN codes, but those are not reported. ACORN captures geodemographic neighborhood characteristics.

Table O2: Selection to the analytical sample

PART 1	(1)<br>Linear probability model	(2)<br>Probit
No father in family	0.031	0.127
	(0.024)	(0.094)
Mother needs qualification for job	0.052***	0.145***
	(0.016)	(0.048)
Father needs qualification for job	0.007	0.023
	(0.011)	(0.035)
SES score	0.035***	0.107***
	(0.008)	(0.026)
SES score missing	-0.078***	-0.318***
	(0.024)	(0.100)
Mother's age	0.003***	0.013***
	(0.001)	(0.004)
Mother's age missing	-0.053	-0.258
	(0.034)	(0.157)
School cohort	0.022**	0.071**
	(0.010)	(0.032)
Has younger sibling	-0.064***	-0.273**
	(0.024)	(0.108)
Has older sibling	-0.011	-0.043
	(0.010)	(0.034)
Father has no qualification	-0.009	-0.041
	(0.016)	(0.057)
Mother has no qualification	-0.060***	-0.269***
	(0.015)	(0.064)
Data on mother's qualification is missing	-0.051	-0.419
	(0.058)	(0.514)
Mother's work: manager	0.003	0.010
	(0.024)	(0.070)
Mother's work: employee	0.007	0.023
	(0.013)	(0.041)
Mother's work: SE with employees	-0.072*	-0.243*
	(0.038)	(0.130)
Mother's work: Foreman	-0.059	-0.188
	(0.089)	(0.292)
Mother's work: SE without employees	-0.284***	
	(0.055)	
Father's work: manager	0.023	0.082
	(0.016)	(0.052)
Father's work: employee	0.033**	0.112**
	(0.014)	(0.046)

PART 2	(1)<br>Linear probability model	(2)<br>Probit
Father's work: SE with employees	0.006	0.031
	(0.021)	(0.070)
Father's work: Foreman	0.003	0.017
	(0.028)	(0.098)
Father's work: SE without employees	0.014	0.067
	(0.133)	(0.632)
Ethnicity: White	0.086***	0.333***
	(0.017)	(0.070)
Ethnicity: missing	0.033	0.139
	(0.076)	(0.330)
Constant	-0.047	-1.783***
	(0.044)	(0.161)
Observations	15,216	15,162
R-squared	0.055	

Source: TEDS (Rimfeld et al., 2019). Sample of those in the first wave. Robust standard errors clustered within twin pairs in parentheses. *** p«0.01, ** p«0.05, * p«0.1 We also control ACORN codes in both models.

First, we use the estimated probabilities of being in the analytical sample from the probit model of Table O2 to create inverse probability weights (IPW). This method ensures that those with higher probability (i.e., those from higher SES backgrounds) get lower weights, so we compensate for them being less likely to drop out. Using these weights, we re-estimate our three most interesting results: our main results as in Table 1 in the main text, the role of parental stereotypical evaluations as in Table 5 in the main text, and the role of male co-twin SAMA as in Table 3 in the main text. These results are reported along with the results of the other two re-weighting methods in Tables O3, O4 and O5 (Block A).

Second, we re-estimate the selection model using a non-parametric machine learning algorithm, random forest. This works by constructing a series of decision trees and predicting the outcome from each series as the modes of predictions (Breiman, 2001). The method offers several advantages. First, as it randomly splits the sample along the explanatory variables, it explicitly models potential non-linear relationships. Thus, if non-linearities are important, it offers better predictions than a probit (where all parameters are linear). Indeed, comparing the (in-sample) predictive power of the probit and the random forest models, the random forest provides almost 50% higher AUC (a measure of predictive power, Kuhn and Johnson (2013)) than the probit (0.99 vs 0.65).[1]

The second advantage of a random forest classification algorithm, besides giving a better prediction, is that it ranks the predictors in terms of their importance (Grömping, 2009), helping us to understand more how selection works. Figure O2 shows the estimated importance measures.

[1] The value of AUC is between 0 and 1, and flipping a coin would produce an AUC of 0.5. As a rule of thumb, the predictive power of a model is considered good if AUC>0.8 and great if AUC>0.9.

Figure O1: The predicted probability of being in the analytical sample (probit model)

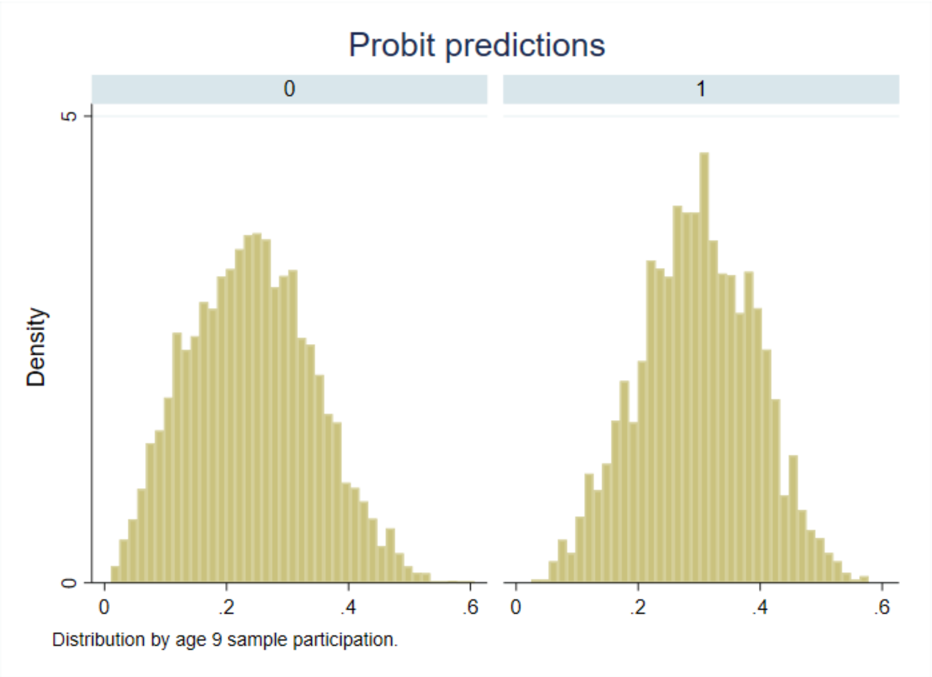

Source: TEDS (Rimfeld et al., 2019). No. of obs: 15,162. Robust standard errors clustered within twin pairs in parentheses. *** p«0.01, ** p«0.05, * p«0.1 The left panel shows the estimated probabilities for those who dropped out, while the right panel for those who are in the analytical sample.

Interestingly, the mother's age and family SES scores are the most important predictors of being selected to the analytical sample, followed by the measures of parents' employment measures.

Figure O2: The importance of predictors in predicting selection to the analytical sample in a random forest model

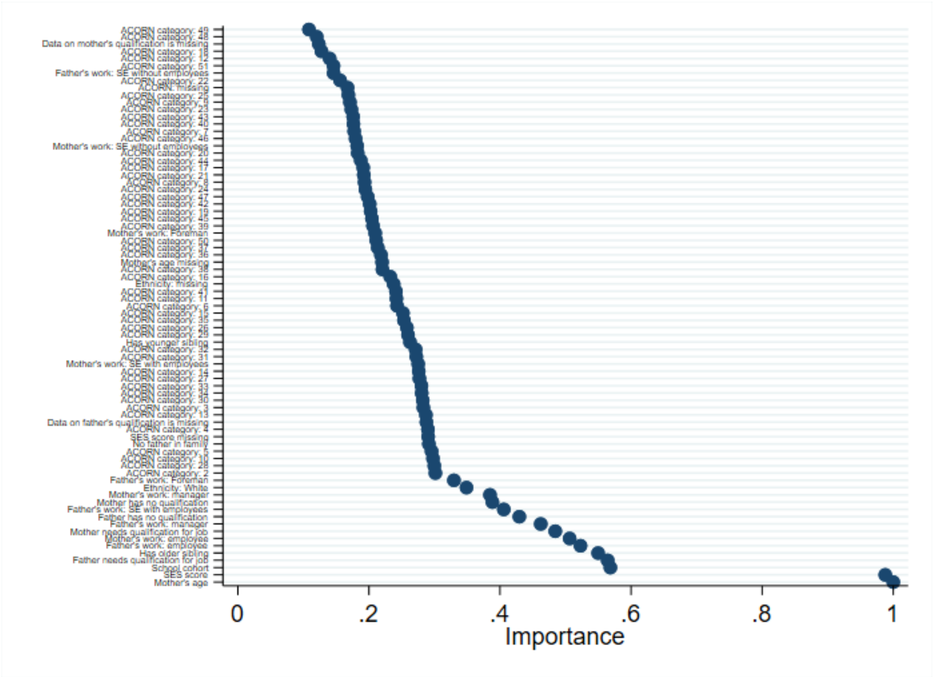

Source: TEDS (Rimfeld et al., 2019). No. of obs: 15,162. Importance shows the relative predictive power of each explanatory variable compared to the predictive power of the most important variable (importance=1). We measure the relative importance of explanatory variables by the Mean Decrease in Gini measure, which captures how well the variable decreases the heterogeneity of subgroups by splitting the sample on a given variable averaged across all decision trees (Friedman et al., 2009).

Third, while the probit model assumes a normal distribution for the predicted probabilities, the random forest does not. Thus, the predictions themselves are quite different (compare Figure O1 and Figure O3). This is useful for us because it is reassuring that our results do not change with either type of re-weighting. Similarly to the probit model, we take the inverse of these probabilities to create IPW's. Our main results re-estimated using these weights are reported in Tables O3, O4 and O5 (Block B).

Lastly, we use entropy balancing (Hainmueller, 2012) to re-weight the analytical sample in a way that the observed characteristics of the sample members follow the distribution of characteristics among those who dropped out. Figure O4 shows the balance of characteristics before and after applying the entropy balanced weights. Using these weights eliminates statistical differences between those in the analytical sample and those who were excluded. Our main results re-estimated using these weights are reported in Tables O3, O4 and O5 (Block C).

Figure O3: The predicted probability of being in the analytical sample (random forest model)

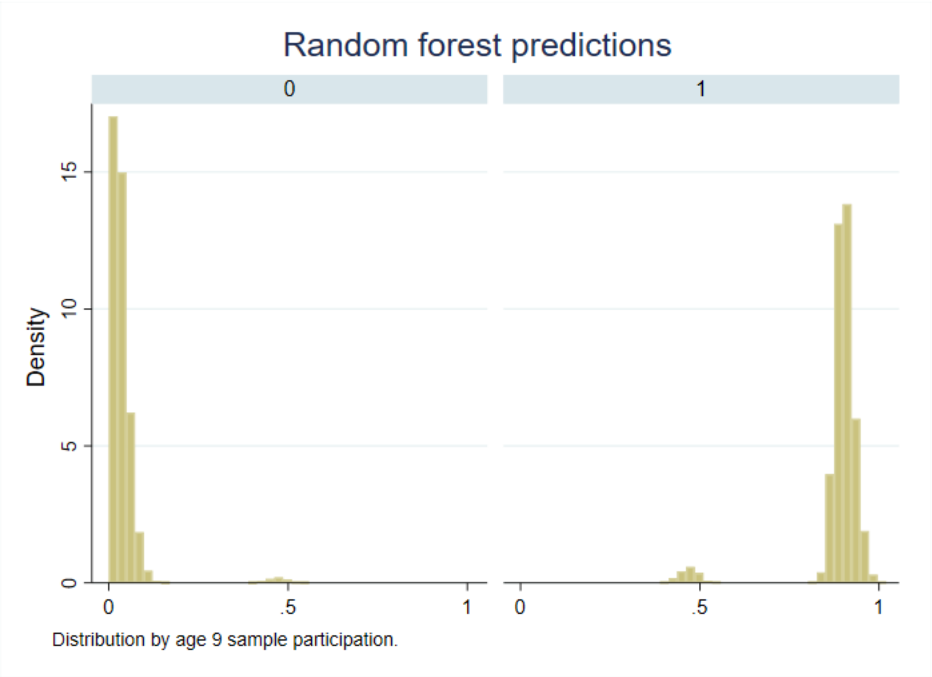

Source: TEDS (Rimfeld et al., 2019). Number of observations: 15,162. The left panel shows the estimated probabilities for those who dropped out, while the right panel for those who are in the analytical sample.

Figure O4: The balance of the analytical sample compared to those who dropped out before and after using entropy-balanced weights)

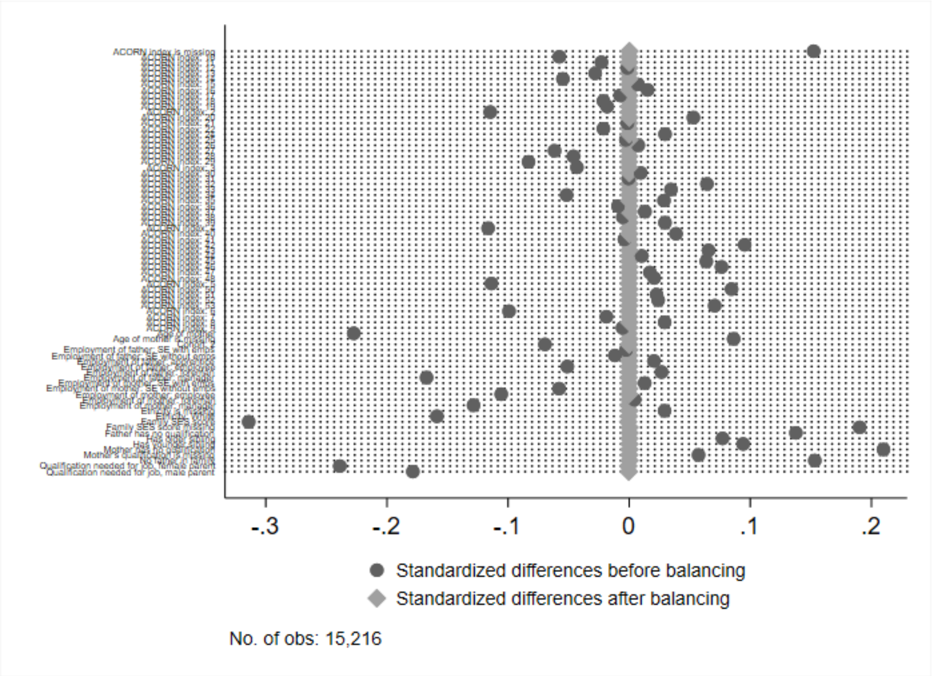

Source: TEDS (Rimfeld et al., 2019). Number of observations: 15,162. Note that after applying the entropy-balanced weights, the standardized differences fall very close to zero; hence the individual points collapse to a vertical line at x=0.

Table O3: The gender gap in mathematics self-assessment (SAMA) - weighted results

	(1)<br>Model 1	(2)<br>Model 2	(3)<br>Model 3	(4)<br>Model 4
Block A: Weighted using probit IPW				
Female	-0.355***	-0.309***	-0.306***	-0.413***
	(0.038)	(0.035)	(0.035)	(0.058)
Block B: Weighted using random forest IPW				
Female	-0.368***	-0.320***	-0.317***	-0.446***
	(0.035)	(0.032)	(0.032)	(0.052)
Block C: Weighted using entropy-balanced weights				
Female	-0.342***	-0.296***	-0.294***	-0.393***
	(0.042)	(0.040)	(0.040)	(0.064)
Observations	3,877	3,877	3,877	3,877
Control variables				
Math levels		Yes	Yes	Yes
Verbal and non-verbal abilities			Yes	Yes
Birth characteristics			Yes	Yes
Twin FE				Yes
Cohort FE	Yes	Yes	Yes	

Notes: Source: TEDS (Rimfeld et al., 2019). Robust standard errors clustered by twin pairs in parentheses. *** p<0.01, ** p<0.05, * p<0.1

Table O4: The role of stereotypically gender-biased parental assessments in the gender gap in SAMA - weighted results

VARIABLES	(1)<br>Model 1	(2)<br>Model 2	(3)<br>Model 3	(4)<br>Model 4	(5)<br>Model 5	(6)<br>Model 6
Block A: Weighted using probit IPW						
Female	-0.306***	-0.306***	-0.015	-0.413***	-0.413***	-0.134**
	(0.035)	(0.035)	(0.042)	(0.058)	(0.058)	(0.068)
Stereotipically assessed person		0.009	0.559***		0.007	0.446***
		(0.038)	(0.049)		(0.059)	(0.086)
Female*stereotipically assessed			-1.085***			-0.889***
			(0.073)			(0.129)
Block B: Weighted using random forest IPW						
Female	-0.316***	-0.316***	-0.019	-0.446***	-0.446***	-0.146**
	(0.032)	(0.032)	(0.037)	(0.052)	(0.052)	(0.061)
Stereotipically assessed person		0.012	0.592***		0.025	0.508***
		(0.034)	(0.043)		(0.051)	(0.076)
Female*stereotipically assessed			-1.108***			-0.956***
			(0.066)			(0.116)
Block C: Weighted using entropy-balanced weights						
Female	-0.294***	-0.293***	-0.003	-0.393***	-0.393***	-0.121
	(0.040)	(0.040)	(0.046)	(0.064)	(0.065)	(0.076)
Stereotipically assessed person		0.016	0.551***		-0.005	0.415***
		(0.043)	(0.057)		(0.064)	(0.092)
Female*stereotipically assessed			-1.082***			-0.875***
			(0.081)			(0.141)
Observations	3,877	3,877	3,877	3,877	3,877	3,877
Twin FE	No	No	No	Yes	Yes	Yes
Cohort FE	Yes	Yes	Yes	No	No	No

Notes: Source: TEDS (Rimfeld et al., 2019). Robust standard errors clustered by twin pairs in parentheses. *** p<0.01, ** p<0.05, * p<0.1 Further control variables: mathematics level at age nine, verbal and non-verbal cognitive skills at age nine, elder twin, heavier twin, and birth weight.

Table O5: The role of co-twin (CT) SAMA - weighted results

VARIABLES	(1)<br>Model 1	(2)<br>Model 2	(3)<br>Model 3	(4)<br>Model 4	(5)<br>Model 4<br>boys	(6)<br>Model 4<br>girls
Block A: probit IPW						
Female	-0.306***	-0.306***	-0.360***	-0.318***		
	(0.034)	(0.034)	(0.039)	(0.037)		
Has a male twin (MT)			-0.138***	-0.113***	-0.114**	-0.104**
			(0.041)	(0.037)	(0.050)	(0.053)
SAMA of CT, age 9, std	0.146***	0.116***	0.176***	0.017	0.017	0.242***
	(0.025)	(0.031)	(0.032)	(0.038)	(0.038)	(0.042)
MT*SAMA of CT			-0.043	0.168***	0.161***	-0.225***
			(0.043)	(0.057)	(0.057)	(0.063)
Female*SAMA of CT		0.056		0.221***		
		(0.041)		(0.055)		
Female*MT*SAMA of CT				-0.390***		
				(0.101)		
Block B: random forest IPW						
Female	-0.324***	-0.324***	-0.382***	-0.341***		
	(0.031)	(0.031)	(0.035)	(0.033)		
Has a male twin (MT)			-0.151***	-0.127***	-0.126***	-0.122***
			(0.036)	(0.033)	(0.045)	(0.047)
SAMA of CT, age 9, std	0.153***	0.127***	0.182***	0.032	0.031	0.246***
	(0.023)	(0.029)	(0.028)	(0.037)	(0.036)	(0.036)
MT*SAMA of CT			-0.034	0.165***	0.161***	-0.208***
			(0.039)	(0.054)	(0.055)	(0.057)
Female*SAMA of CT		0.048		0.209***		
		(0.037)		(0.050)		
Female*MT*SAMA of CT				-0.371***		
				(0.094)		
Block C: entropy balanced weights						
Female	-0.295***	-0.294***	-0.346***	-0.304***		
	(0.038)	(0.038)	(0.043)	(0.041)		
Has a male twin (MT)			-0.130***	-0.104**	-0.119**	-0.082
			(0.045)	(0.041)	(0.058)	(0.057)
SAMA of CT, age 9, std	0.140***	0.111***	0.168***	0.005	0.008	0.235***
	(0.027)	(0.034)	(0.035)	(0.041)	(0.041)	(0.046)
MT*SAMA of CT			-0.041	0.175***	0.166***	-0.235***
			(0.047)	(0.062)	(0.063)	(0.069)
Female*SAMA of CT		0.053		0.226***		
		(0.045)		(0.060)		
Female*MT*SAMA of CT				-0.404***		
				(0.111)		
Observations	3,722	3,722	3,722	3,722	1,707	2,015

Notes: Source: TEDS (Rimfeld et al., 2019). Robust standard errors clustered by twin pairs in parentheses. *** p<0.01, ** p<0.05, * p<0.1 Further control variables: mathematics level at age nine, verbal and non-verbal cognitive skills at age nine, elder twin, heavier twin, birth weight and cohort FE.

O2 Supporting information

Table O6: The gender gap in SAMA, fully interacted main model, age nine

VARIABLES	(1)<br>Model 1	(2)<br>Model 2	(3)<br>Model 3	(4)<br>Model 4
Female	-0.376***	-0.325***	-0.174	-0.188
	(0.034)	(0.032)	(0.160)	(0.262)
Math level, age 9		0.387***	0.326***	0.356***
		(0.022)	(0.018)	(0.032)
Female*math level		-0.030		
		(0.033)		
Verbal abilities, age 9			0.074***	0.139***
			(0.026)	(0.049)
Female*verbal abilities			-0.038	-0.101*
			(0.036)	(0.058)
Non-verbal abilities, age 9			0.069**	0.123***
			(0.027)	(0.044)
Female*nonverbal abilities			-0.016	0.010
			(0.037)	(0.056)
Elder twin			0.050	-0.003
			(0.038)	(0.042)
Female*elder twin			-0.024	0.065
			(0.054)	(0.062)
Heavier twin at birth			0.024	0.028
			(0.042)	(0.060)
Female*heavier twin			0.033	0.010
			(0.058)	(0.073)
Birthweight, gramms			0.000*	0.000
			(0.000)	(0.000)
Female*birthweight			-0.000	-0.000
			(0.000)	(0.000)
Constant	0.182***	0.116***	-0.140	-0.140
	(0.035)	(0.032)	(0.116)	(0.287)
Observations	3,877	3,877	3,877	3,877
R-squared	0.036	0.165	0.175	0.167
Twin FE	No	No	No	Yes
Cohort FE	Yes	Yes	Yes	No

Source: TEDS (Rimfeld et al., 2019). Robust standard errors clustered by twin pairs in parentheses. *** p<0.01, ** p<0.05, * p<0.1

Table O7: The heterogeneity of the gender gap in SAMA along the distribution of abilities, age nine (same control variables as in Table 1 in the main text)

VARIABLES	(1)<br>Model 3	(2)<br>Model 4	(3)<br>Model 3	(4)<br>Model 4
Female = 1	-0.351***	-0.482***	-0.333***	-0.443***
	(0.039)	(0.063)	(0.047)	(0.072)
Math grade category = 1	-0.231***	-0.154		
	(0.076)	(0.117)		
Math grade category = 2	0.057	0.086		
	(0.101)	(0.150)		
Math grade category*female = 1	0.035	0.109		
	(0.069)	(0.105)		
Math grade category*female = 2	0.121	0.094		
	(0.101)	(0.152)		
High ability = 1			0.078	0.156*
			(0.060)	(0.087)
High ability*female = 1			0.019	-0.009
			(0.061)	(0.091)
Constant	-0.028	0.046	-0.104	-0.056
	(0.091)	(0.240)	(0.093)	(0.242)
Observations	3,877	3,877	3,877	3,877
R-squared	0.176	0.165	0.174	0.166
Twin FE	No	Yes	No	Yes
Cohort FE	Yes	No	Yes	No

Source: TEDS (Rimfeld et al., 2019). Control variables: mathematics level at age nine, verbal and non-verbal cognitive skills at age nine, elder twin, heavier twin, and birth weight. Robust standard errors clustered by twin pairs in parentheses. *** p<0.01, ** p<0.05, * p<0.1

Table O8: The heterogeneity of the gender gap in SAMA along the distribution of abilities, age nine (without controlling for abilities)

	(1)	(2)	(3)	(4)
VARIABLES	Model 3	Model 4	Model 3	Model 4
Female = 1	-0.363***	-0.483***	-0.347***	-0.473***
	(0.040)	(0.065)	(0.049)	(0.075)
Math grade category = 1	0.452***	0.499***		
	(0.045)	(0.078)		
Math grade category = 2	-0.712***	-0.629***		
	(0.074)	(0.117)		
Math grade category*female = 1	0.025	0.070		
	(0.070)	(0.108)		
Math grade category*female = 2	0.161	0.098		
	(0.103)	(0.156)		
High ability = 1			0.376***	0.407***
			(0.046)	(0.073)
High ability*female = 1			-0.027	-0.072
			(0.065)	(0.095)
Constant	-0.031	-0.044	-0.332***	-0.242
	(0.092)	(0.245)	(0.099)	(0.257)
Observations	3,877	3,877	3,877	3,877
R-squared	0.145	0.129	0.074	0.085
Twin FE	No	Yes	No	Yes
Cohort FE	Yes	No	Yes	No

Source: TEDS (Rimfeld et al., 2019). Control variables: elder twin, heavier twin, and birth weight. Robust standard errors clustered by twin pairs in parentheses. *** p<0.01, ** p<0.05, * p<0.1

Table O9: The gender gap in mathematics levels, age nine

VARIABLES	(1)<br>Model 1	(2)<br>Model 3	(3)<br>Model 4	(4)<br>Model 5	(5)<br>Model 6	(6)<br>Model 7
Female	-0.133***	-0.106***	-0.174***	-0.129***	-0.160***	-0.128***
	(0.034)	(0.030)	(0.041)	(0.028)	(0.045)	(0.028)
Has a male twin				-0.059**	-0.092**	-0.059**
				(0.028)	(0.047)	(0.028)
Has brother						-0.041
						(0.036)
Has sister						-0.022
						(0.036)
Verbal abilities, age 9		0.214***	0.202***	0.214***	0.213***	0.212***
		(0.017)	(0.026)	(0.017)	(0.017)	(0.017)
Non-verbal abilities, age 9		0.302***	0.243***	0.303***	0.303***	0.302***
		(0.017)	(0.026)	(0.017)	(0.017)	(0.017)
Elder twin		0.015	0.013	0.015	0.014	0.015
		(0.020)	(0.020)	(0.020)	(0.020)	(0.020)
Heavier twin at birth		0.053**	0.091***	0.047**	0.048**	0.045**
		(0.023)	(0.033)	(0.023)	(0.023)	(0.023)
Birthweight, gramms		0.000***	-0.000	0.000***	0.000***	0.000***
		(0.000)	(0.000)	(0.000)	(0.000)	(0.000)
Female*male twin					0.064	
					(0.069)	
Constant	0.148***	-0.219***	0.130	-0.181**	-0.153*	-0.179**
	(0.036)	(0.081)	(0.191)	(0.082)	(0.089)	(0.082)
Observations	4,309	4,309	4,309	4,309	4,309	4,309
R-squared	0.005	0.202	0.129	0.203	0.203	0.203
Twin FE	No	No	Yes	No	No	No
Cohort FE	Yes	Yes	No	Yes	Yes	Yes

Source: TEDS (Rimfeld et al., 2019). Robust standard errors clustered by twin pairs in parentheses. *** p<0.01, ** p<0.05, * p<0.1

Table O10: The gender gap in self-assessed English abilities, age nine

VARIABLES	(1)<br>Model 1	(2)<br>Model 2	(3)<br>Model 3	(4)<br>Model 4
Female	0.224***	0.132***	0.141***	0.231***
	(0.035)	(0.032)	(0.032)	(0.053)
English level, age 9		0.356***	0.333***	0.382***
		(0.018)	(0.020)	(0.038)
Verbal abilities, age 9			0.095***	0.119***
			(0.019)	(0.038)
Non-verbal abilities, age 9			-0.032	0.007
			(0.019)	(0.037)
Constant	-0.113***	-0.106***	-0.054	-0.203
	(0.034)	(0.031)	(0.086)	(0.229)
Observations	3,877	3,877	3,877	3,877
R-squared	0.012	0.128	0.135	0.107
Twin FE	No	No	No	Yes
Cohort FE	Yes	Yes	Yes	No

Source: TEDS (Rimfeld et al., 2019). Robust standard errors clustered by twin pairs in parentheses. *** p<0.01, ** p<0.05, * p<0.1

Table O11: The gender gap in English levels, age nine

VARIABLES	(1)<br>Model 1	(2)<br>Model 2	(3)<br>Model 3	(4)<br>Model 3 OS subsample	(5)<br>Model 4
Female	0.256***	0.350***	0.351***	0.353***	0.331***
	(0.035)	(0.023)	(0.023)	(0.032)	(0.031)
Math level, age 9		0.729***	0.683***	0.670***	0.531***
		(0.013)	(0.014)	(0.024)	(0.022)
Verbal abilities, age 9			0.112***	0.143***	0.114***
			(0.013)	(0.022)	(0.022)
Non-verbal abilities, age 9			0.023*	0.035	0.036*
			(0.013)	(0.023)	(0.019)
Elder twin			-0.020	-0.060*	-0.023
			(0.016)	(0.031)	(0.016)
Heavier twin at birth			0.002	0.024	0.002
			(0.018)	(0.036)	(0.025)
Birthweight, gramms			0.000	0.000	0.000
			(0.000)	(0.000)	(0.000)
Constant	-0.019	-0.145***	-0.198***	-0.134	-0.323**
	(0.037)	(0.024)	(0.065)	(0.115)	(0.133)
Observations	3,877	3,877	3,877	1,186	3,877
R-squared	0.018	0.553	0.566	0.587	0.399
Twin FE	No	No	No	No	Yes
Cohort FE	Yes	Yes	Yes	Yes	No

Robust standard errors in parentheses
*** p<0.01, ** p<0.05, * p<0.1

Source: TEDS (Rimfeld et al., 2019). Robust standard errors clustered by twin pairs in parentheses. *** p<0.01, ** p<0.05, * p<0.1

Table O12: The gender gap in parental assessment of their children's mathematics abilities

VARIABLES	(1)<br>Model 1	(2)<br>Model 2	(3)<br>Model 3	(4)<br>Model 3 OS subsample	(5)<br>Model 4
Female	-0.289***	-0.212***	-0.209***	-0.400***	-0.417***
	(0.037)	(0.030)	(0.029)	(0.043)	(0.042)
Math level, age 9		0.591***	0.517***	0.490***	0.499***
		(0.016)	(0.017)	(0.029)	(0.028)
Verbal abilities, age 9			0.082***	0.047	0.094***
			(0.017)	(0.031)	(0.026)
Non-verbal abilities, age 9			0.110***	0.108***	0.146***
			(0.018)	(0.034)	(0.026)
Elder twin			0.034*	0.016	0.034*
			(0.020)	(0.042)	(0.019)
Heavier twin at birth			0.021	0.079	0.020
			(0.023)	(0.049)	(0.031)
Birthweight, gramms			0.000***	-0.000	0.000
			(0.000)	(0.000)	(0.000)
Constant	0.110***	0.007	-0.251***	0.082	-0.002
	(0.039)	(0.030)	(0.080)	(0.145)	(0.180)
Observations	3,877	3,877	3,877	1,186	3,877
R-squared	0.022	0.348	0.370	0.367	0.355
Twin FE	No	No	No	No	Yes
Cohort FE	Yes	Yes	Yes	Yes	No

Robust standard errors in parentheses
*** p<0.01, ** p<0.05, * p<0.1

Notes: Source: TEDS (Rimfeld et al., 2019). Robust standard errors clustered by twin pairs in parentheses. *** p<0.01, ** p<0.05, * p<0.1

Table O13: The gender gap in teachers' assessments of children's mathematics abilities

VARIABLES	(1)<br>Model 1	(2)<br>Model 2	(3)<br>Model 3	(4)<br>Model 3 OS subsample	(5)<br>Model 4
Female	-0.224***	-0.120***	-0.123***	-0.147***	-0.159***
	(0.036)	(0.022)	(0.022)	(0.031)	(0.031)
Math level, age 9		0.804***	0.768***	0.761***	0.721***
		(0.012)	(0.013)	(0.024)	(0.020)
Verbal abilities, age 9			0.017	0.017	0.058***
			(0.013)	(0.023)	(0.020)
Non-verbal abilities, age 9			0.078***	0.115***	0.056***
			(0.013)	(0.023)	(0.018)
Elder twin			0.012	-0.015	0.019
			(0.015)	(0.030)	(0.016)
Heavier twin at birth			0.010	0.042	0.047*
			(0.018)	(0.035)	(0.026)
Birthweight, gramms			0.000	0.000	-0.000
			(0.000)	(0.000)	(0.000)
Constant	0.109***	-0.030	-0.084	-0.093	0.221
	(0.040)	(0.024)	(0.061)	(0.109)	(0.147)
Observations	3,877	3,877	3,877	1,186	3,877
R-squared	0.013	0.614	0.620	0.643	0.535
Twin FE	No	No	No	No	Yes
Cohort FE	Yes	Yes	Yes	Yes	No

Robust standard errors in parentheses
*** p<0.01, ** p<0.05, * p<0.1

Notes: Source: TEDS (Rimfeld et al., 2019). Robust standard errors clustered by twin pairs in parentheses. *** p<0.01, ** p<0.05, * p<0.1

Table O14: The role of parental and teachers' assessments in the gender gap in SAMA, FE models

	(1)	(2)	(3)	(4)	(5)	(6)
VARIABLES	Model 1	Model 2	Model 3	Model 4	Model 5	Model 6
Female	-0.218***	-0.005	-0.401***	-0.134	-0.210***	0.066
	(0.048)	(0.231)	(0.050)	(0.211)	(0.048)	(0.238)
Parental assessment of Math	0.591***	0.619***			0.564***	0.572***
	(0.039)	(0.050)			(0.040)	(0.054)
Female*parental assessment		-0.053				-0.015
		(0.054)				(0.065)
Teachers' assessment of Math			0.351***	0.392***	0.140***	0.173***
			(0.050)	(0.059)	(0.047)	(0.057)
Female*teachers' assessment				-0.079		-0.064
				(0.058)		(0.063)
Constant	-2.296***	-2.414***	-1.214***	-1.352***	-2.689***	-2.832***
	(0.254)	(0.296)	(0.278)	(0.306)	(0.274)	(0.316)
Observations	3,877	3,877	3,877	3,877	3,877	3,877
R-squared	0.297	0.298	0.188	0.189	0.301	0.301
Twin FE	Yes	Yes	Yes	Yes	Yes	Yes
Cohort FE	No	No	No	No	No	No

Source: TEDS (Rimfeld et al., 2019). Robust standard errors clustered by twin pairs in parentheses. *** p<0.01, ** p<0.05, * p<0.1 Further control variables: mathematics level at age nine, verbal and non-verbal cognitive skills at age nine, elder twin, heavier twin, and birth weight.

Table O15: The gender gap in parent's stereotypical assessments of their children's math abilities

VARIABLES	(1)<br>Model 1	(2)<br>Model 2	(3)<br>Model 3	(4)<br>Model 3 OS subsample	(5)<br>Model 4
Female	-0.002	0.005	0.004	-0.042	-0.035
	(0.016)	(0.017)	(0.017)	(0.030)	(0.030)
Math level, age 9		0.051***	0.045***	0.033***	0.070***
		(0.007)	(0.008)	(0.013)	(0.015)
Verbal abilities, age 9			0.009	0.014	0.014
			(0.009)	(0.015)	(0.016)
Non-verbal abilities, age 9			0.008	-0.023	0.004
			(0.009)	(0.016)	(0.016)
Elder twin			0.001	-0.032	0.002
			(0.012)	(0.029)	(0.013)
Heavier twin at birth			-0.008	-0.017	-0.031
			(0.014)	(0.031)	(0.021)
Birthweight, gramms			0.000	0.000	0.000
			(0.000)	(0.000)	(0.000)
Constant	0.264***	0.256***	0.245***	0.341***	0.204*
	(0.016)	(0.016)	(0.040)	(0.067)	(0.116)
Observations	3,877	3,877	3,877	1,186	3,877
R-squared	0.000	0.012	0.013	0.010	0.019
Twin FE	No	No	No	No	Yes
Cohort FE	Yes	Yes	Yes	Yes	No

Robust standard errors in parentheses
*** p<0.01, ** p<0.05, * p<0.1

Notes: Source: TEDS (Rimfeld et al., 2019). The outcome is a binary variable that captures whether parents' assessment of their children's mathematics abilities is stereotypically gender-biased. It takes the value one if they either: overestimate their son in math or underestimate their daughter in math. The variable is child-specific and may vary within twins/families. Robust standard errors clustered by twin pairs in parentheses. *** p<0.01, ** p<0.05, * p<0.1

Table O16: The role of the mathematics level of co-twin (CT) in the gender gap in SAMA

VARIABLES	(1) Model 1	(2) Model 2	(3) Model 3	(4) Model 4	(5) Model 4 boys	(6) Model 4 girls
Female	-0.322***	-0.321***	-0.350***	-0.347***		
	(0.032)	(0.032)	(0.032)	(0.033)		
Math level of CT, age 9	-0.044**	-0.038	-0.049*	-0.080**	-0.087**	-0.024
	(0.018)	(0.024)	(0.025)	(0.038)	(0.040)	(0.033)
Female*Math level of CT		-0.013		0.047		
		(0.033)		(0.049)		
Has a male twin (MT)			-0.074**	-0.072**	-0.084*	-0.054
			(0.032)	(0.033)	(0.046)	(0.049)
MT*Math level of CT			0.014	0.066	0.064	-0.040
			(0.033)	(0.046)	(0.047)	(0.049)
Female*MT*Math level of CT				-0.103		
				(0.066)		
Constant	-0.065	-0.065	-0.017	-0.020	-0.083	-0.299**
	(0.089)	(0.089)	(0.091)	(0.091)	(0.124)	(0.123)
Observations	3,877	3,877	3,877	3,877	1,781	2,096
R-squared	0.175	0.175	0.176	0.177	0.187	0.116
Twin FE	No	No	No	No	No	No
Cohort FE	Yes	Yes	Yes	Yes	Yes	Yes

Source: TEDS (Rimfeld et al., 2019). Robust standard errors clustered by twin pairs in parentheses. *** p<0.01, ** p<0.05, * p<0.1 Further control variables: mathematics level at age nine, verbal and non-verbal cognitive skills at age nine, elder twin, heavier twin, and birth weight.

Table O17: The role of co-twin (CT) SAMA - co-twin SAMA as binary variable

VARIABLES	(1) Model 1	(2) Model 2	(3) Model 3	(4) Model 4	(5) Model 4 boys	(6) Model 4 girls
Female	-0.329***	-0.306***	-0.373***	-0.339***		
	(0.032)	(0.036)	(0.034)	(0.036)		
Has a male twin (MT)			-0.097***	-0.083**	-0.158***	0.002
			(0.037)	(0.036)	(0.051)	(0.055)
Confident twin	0.224***	0.287***	0.321***	0.168	0.127	0.403***
	(0.043)	(0.056)	(0.065)	(0.103)	(0.107)	(0.080)
Male twin*confident twin			-0.123	0.167	0.234*	-0.485***
			(0.081)	(0.118)	(0.128)	(0.118)
Female*confident twin		-0.122		0.209*		
		(0.078)		(0.127)		
Female*male twin*confident twin				-0.569***		
				(0.162)		
Constant	-0.101	-0.116	-0.042	-0.072	-0.070	-0.361***
	(0.089)	(0.090)	(0.091)	(0.091)	(0.122)	(0.123)
Observations	3,722	3,722	3,722	3,722	1,707	2,015
R-squared	0.181	0.182	0.185	0.188	0.198	0.129
Twin FE	No	No	No	No	No	No
Cohort FE	Yes	Yes	Yes	Yes	Yes	Yes

Notes: Source: TEDS (Rimfeld et al., 2019). Robust standard errors clustered within twin pairs in parentheses. *** p<0.01, ** p<0.05, * p<0.1 The variable "Confident twin" is a binary variable that equals 1 if co-twin's SAMA belongs to the top 20% of the SAMA distribution and 0 otherwise. Further control variables: mathematics level at age nine, verbal and non-verbal cognitive skills at age nine, elder twin, heavier twin, and birth weight. CT refers to co-twins.

Table O18: The gender gap in self-assessed English abilities - the role of co-twins (CT)

	(1)	(2)	(3)	(4)	(5)	(6)
					Model 4	Model 4
VARIABLES	Model 1	Model 2	Model 3	Model 4	boys	girls
Female	0.248***	0.248***	0.297***	0.270***		
	(0.031)	(0.031)	(0.036)	(0.033)		
Has a male twin (MT)			0.127***	0.110***	0.109**	0.116***
			(0.036)	(0.033)	(0.050)	(0.041)
Self-assessed English ability of CT	0.181***	0.173***	0.223***	0.074	0.071	0.273***
	(0.024)	(0.034)	(0.033)	(0.049)	(0.049)	(0.040)
Female*self-assessed English ability of CT		0.015		0.193***		
		(0.042)		(0.063)		
MT*self-assessed English ability of CT			-0.070*	0.142**	0.139**	-0.236***
			(0.042)	(0.065)	(0.065)	(0.051)
Female*MT*self-assessed English ability of CT				-0.376***		
				(0.098)		
Constant	-0.140*	-0.139*	-0.224***	-0.196**	-0.201	0.084
	(0.078)	(0.078)	(0.082)	(0.080)	(0.124)	(0.094)
Observations	3,876	3,876	3,876	3,876	1,781	2,095
R-squared	0.112	0.112	0.116	0.123	0.114	0.113
Twin FE	No	No	No	No	No	No
Cohort FE	Yes	Yes	Yes	Yes	Yes	Yes

Source: TEDS (Rimfeld et al., 2019). Robust standard errors clustered by twin pairs in parentheses. *** p«0.01, ** p«0.05, * p«0.1 Further control variables: mathematics level at age nine, verbal and non-verbal cognitive skills at age nine, elder twin, heavier twin, and birth weight.

Table O19: The gender gap in self-assessed physical abilities - the role of co-twins (CT)

VARIABLES	(1)<br>Model 1	(2)<br>Model 2	(3)<br>Model 3	(4)<br>Model 4	(5)<br>Model 4<br>boys	(6)<br>Model 4<br>girls
Female	-0.058*	-0.058*	-0.055	-0.048		
	(0.031)	(0.031)	(0.037)	(0.033)		
Has a male twin (MT)			0.009	0.009	0.014	-0.004
			(0.038)	(0.033)	(0.047)	(0.045)
Self-assessed physical ability of CT	0.236***	0.230***	0.246***	0.083*	0.085*	0.305***
	(0.027)	(0.037)	(0.033)	(0.050)	(0.050)	(0.040)
Female*self-assessed physical ability of CT		0.012		0.223***		
		(0.044)		(0.064)		
MT*self-assessed physical ability of CT			-0.021	0.220***	0.218***	-0.230***
			(0.045)	(0.068)	(0.068)	(0.064)
Female*MT*self-assessed physical ability of CT				-0.453***		
				(0.116)		
Constant	-0.107	-0.107	-0.111	-0.117	-0.070	-0.205**
	(0.076)	(0.076)	(0.082)	(0.080)	(0.123)	(0.093)
Observations	3,853	3,853	3,853	3,853	1,767	2,086
R-squared	0.062	0.062	0.062	0.073	0.065	0.079
Twin FE	No	No	No	No	No	No
Cohort FE	Yes	Yes	Yes	Yes	Yes	Yes

Source: TEDS (Rimfeld et al., 2019). Robust standard errors clustered by twin pairs in parentheses. *** p<0.01, ** p<0.05, * p<0.1 Further control variables: mathematics level at age nine, verbal and non-verbal cognitive skills at age nine, elder twin, heavier twin, and birth weight.

Table O20: The role of parental education, age nine

VARIABLES	(1) SAMA	(2) SAMA	(3) SAMA	(4) Math level	(5) Math level	(6) Math level
Female	-0.327***	-0.183*	-0.274*	-0.128***	0.113	0.047
	(0.032)	(0.101)	(0.147)	(0.034)	(0.109)	(0.142)
Parental education						
High-grade CSE/GCSE	-0.128**	-0.053		0.189***	0.339***	
	(0.060)	(0.086)		(0.066)	(0.101)	
A-level or below degree	-0.090	-0.002		0.373***	0.542***	
	(0.061)	(0.087)		(0.067)	(0.102)	
Degree	-0.129**	-0.032		0.667***	0.809***	
	(0.061)	(0.088)		(0.066)	(0.099)	
Female*High-grade CSE/GCSE		-0.136	-0.191		-0.265**	-0.222
		(0.116)	(0.179)		(0.125)	(0.167)
Female*A-level or below degree		-0.167	-0.217		-0.298**	-0.274*
		(0.116)	(0.178)		(0.127)	(0.162)
Female*Degree		-0.178	-0.188		-0.247**	-0.254
		(0.115)	(0.168)		(0.124)	(0.161)
Math level, age 9						
Constant	0.070	-0.045	0.042	-0.179***	-0.315***	0.184***
	(0.098)	(0.114)	(0.237)	(0.066)	(0.090)	(0.024)
Observations	3,863	3,863	3,863	3,863	3,863	3,863
R-squared	0.173	0.175	0.163	0.060	0.062	0.013
Twin FE	No	No	Yes	No	No	Yes
Cohort FE	Yes	Yes	No	Yes	Yes	No

Notes: Source: TEDS (Rimfeld et al., 2019). Robust standard errors clustered by twin pairs in parentheses. *** p<0.01, ** p<0.05, * p<0.1. Further control variables: mathematics level at age nine, verbal and non-verbal cognitive skills at age nine, elder twin, heavier twin, and birth weight.

Table O21: The role of maternal characteristics in the gender gap in SAMA, age nine

VARIABLES	(1)<br>Model 1	(2)<br>Model 2	(3)<br>Model 3	(4)<br>Model 4	(5)<br>Model 5	(6)<br>Model 6
Female	-0.290***	-0.320***	-0.318***	-0.385***	-0.437***	-0.422***
	(0.042)	(0.033)	(0.037)	(0.070)	(0.055)	(0.061)
Mother has A-levels or above	0.016					
	(0.046)					
Female*Mother has A-levels or above	-0.081			-0.145		
	(0.063)			(0.101)		
Mother has managerial job		0.002				
		(0.072)				
Female*Mother has managerial job		-0.039			-0.083	
		(0.105)			(0.150)	
Mother needs qualification			-0.027			
			(0.050)			
Female*Mother needs qualification			-0.030			-0.101
			(0.071)			(0.109)
Constant	-0.074	-0.066	-0.058	0.027	0.034	0.033
	(0.090)	(0.089)	(0.089)	(0.236)	(0.236)	(0.236)
Observations	3,877	3,877	3,877	3,877	3,877	3,877
R-squared	0.174	0.174	0.174	0.165	0.164	0.164
Twin FE	No	No	No	Yes	Yes	Yes
Cohort FE	Yes	Yes	Yes	No	No	No

Notes: Source: TEDS (Rimfeld et al., 2019). Robust standard errors clustered by twin pairs in parentheses. *** p<0.01, ** p<0.05, * p<0.1. Further control variables: mathematics level at age nine, verbal and non-verbal cognitive skills at age nine, elder twin, heavier twin, and birth weight.

Table O22: The role of stereotypical parental assessments in the twin peer effects in SAMA, age nine

VARIABLES	(1)<br>Not OE boys	(2)<br>OE boys	(3)<br>Not UE girls	(4)<br>UE girls
Has a male twin (MT)	-0.074	-0.017	-0.043	-0.251***
	(0.054)	(0.067)	(0.055)	(0.083)
SAMA of CT, age 9, std	0.046	0.040	0.209***	0.245***
	(0.048)	(0.051)	(0.041)	(0.063)
MT*SAMA of CT	0.167**	-0.079	-0.198***	-0.180*
	(0.065)	(0.070)	(0.064)	(0.097)
Constant	-0.170	0.535***	-0.169	-0.381*
	(0.131)	(0.195)	(0.126)	(0.218)
Observations	1,256	451	1,489	526
R-squared	0.270	0.099	0.214	0.126
Twin FE	No	No	No	No
Cohort FE	Yes	Yes	Yes	Yes

Notes: Source: TEDS (Rimfeld et al., 2019). Robust standard errors clustered by twin pairs in parentheses. *** p<0.01, ** p<0.05, * p<0.1. Further control variables: mathematics level at age nine, verbal and non-verbal cognitive skills at age nine, elder twin, heavier twin, and birth weight.

References

Adamecz-Völgyi, A., Shure, N., 2022. The gender gap in top jobs – the role of overconfidence. Labour Economics , 102283URL: https://linkinghub.elsevier.com/retrieve/pii/S0927537122001737, doi:10.1016/j.labeco.2022.102283.

Breiman, L., 2001. Random Forests. Machine Learning 45, 5–32. URL: https://link.springer.com/article/10.1023/A:1010933404324, doi:10.1023/A:1010933404324.

Friedman, J., Hastie, T., Tibshirani, R., 2009. The Elements of Statistical Learning: Data Mining, Inference, and Prediction. Second edition ed., Springer.

Gillen, B., Snowberg, E., Yariv, L., 2019. Experimenting with Measurement Error: Techniques with Applications to the Caltech Cohort Study. Journal of Political Economy 127, 1826–1863. URL: https://www.journals.uchicago.edu/doi/full/10.1086/701681, doi:10.1086/701681. publisher: The University of Chicago Press.

Grömping, U., 2009. Variable Importance Assessment in Regression: Linear Regression versus Random Forest. The American Statistician 63, 308–319. URL: https://doi.org/10.1198/tast.2009.08199, doi:10.1198/tast.2009.08199.

Hainmueller, J., 2012. Entropy Balancing for Causal Effects: A Multivariate Reweighting Method to Produce Balanced Samples in Observational Studies. Political Analysis 20, 25–46. URL: https://www.cambridge.org/core/product/identifier/S1047198700012997/type/journal_article, doi:10.1093/pan/mpr025.

Kuhn, M., Johnson, K., 2013. Applied Predictive Modeling. Springer Science & Business Media. Google-Books-ID: xYRDAAAAQBAJ.

Rimfeld, K., Malanchini, M., Spargo, T., Spickernell, G., Selzam, S., McMillan, A., Dale, P.S., Eley, T.C., Plomin, R., 2019. Twins Early Development Study: A Genetically Sensitive Investigation into Behavioral and Cognitive Development from Infancy to Emerging Adulthood. Twin Research and Human Genetics 22, 508–513. URL: https://www.cambridge.org/core/journals/twin-research-and-human-genetics/article/twins-early-development-study-a-genetically-sensitive-investigation-into-behavioral-and-cognitive-development-from-infancy-to-emerging-adulthood/E3E789B968536AB5DD5CAB63883E9CC2#, doi:10.1017/thg.2019.56. publisher: Cambridge University Press.
